# Assessment of General Toxicity of the *Glycyrrhiza* New Variety Extract in Rats

**DOI:** 10.3390/plants10061126

**Published:** 2021-06-01

**Authors:** Dong-Gu Kim, Jeonghoon Lee, Wonnam Kim, Hyo-Jin An, Jong-Hyun Lee, Jaeki Chang, Sa-Haeng Kang, Young-Jae Song, Yong-Deok Jeon, Jong-Sik Jin

**Affiliations:** 1 Department of Oriental Medicine Resources, Jeonbuk National University, 79 Gobong-ro, Iksan 54596, Jeollabuk-do, Korea; kdg2409@naver.com (D.-G.K.); kangsh@jbnu.ac.kr (S.-H.K.); dudwoid@naver.com (Y.-J.S.); 2 Herbal Crop Research Division, National Institute of Horticultural and Herbal Science, Rural Development Administration, 92 Bisan-ro, Eumseong 27709, Chungbuk, Korea; artemisia@korea.kr; 3 Cnh Center for Cancer Research, 462 Bongeunsa-ro, Gangnam-gu, Seoul 06154, Korea; eb75lab@gmail.com; 4 Department of Pharmacology, College of Korean Medicine, Sangji University, 83 Sangjidae-gil, Wonju-si 26339, Gangwon-do, Korea; sangjipharm@gmail.com; 5 Department of Pharmacy, College of Pharmacy, Dongduk Women’s University, 60 Hwarang-ro 13-gil, Seongbuk-gu, Seoul 02748, Korea; naturalmed@dongduk.ac.kr; 6 Crop Production & Physiology Division, National Institute of Crop Science, Rural Development Administration, 181 Hyeoksin-ro, Wanju 55365, Jeollabuk-do, Korea; changjk@korea.kr; 7 Department of Korean Pharmacy, College of Pharmacy, Woosuk University, 443 Samrye-ro, Samrye-eup, Wanju-gun 55338, Jeollabuk-do, Korea

**Keywords:** *Glycyrrhiza*, Wongam, single oral dose toxicity study, 4-week repeated oral dose toxicity study, 13-week repeated oral dose toxicity study

## Abstract

The *Glycyrrhiza* radix (Licorice) is one of the most commonly used medicinal plants in Asian countries, such as China, India, and Korea. It has been traditionally used to treat many diseases, including cough, cold, asthma, fatigue, gastritis, and respiratory tract infections. A *Glycyrrhiza* new variety, Wongam (WG), has been developed by the Korea Rural Development Administration and revealed pharmacological effects. However, the potential adverse effects of WG have not been revealed yet. This study evaluates the general toxicity of the WG extract through a single and repeated oral dose toxicity study in Sprague-Dawley rats. After single oral dose administration, no significant toxicological changes or mortality was observed up to 5000 mg/kg. Over a 4-week repeated oral dose toxicity study, no adverse effects and target organs were observed up to 5000 mg/kg/day. Over a 13-week repeated oral dose toxicity study, no mortality or toxicological changes involving ophthalmology, water consumption, or hematology were observed up to 5000 mg/kg/day. Although other parameters were changed, the alterations in question were not considered toxicologically significant, since responses remained within normal ranges and were not dose-dependent. In conclusion, the no-observed-adverse-effect level (NOAEL) of WG was higher than 5000 mg/kg/day, and no target organs were identified in rats.

## 1. Introduction

Licorice, a perennial plant belonging to the Leguminosae family, is widely distributed in the deserts and on the grasslands of Asia, Europe, and the Americas [1]. It has been used in traditional medicines and folk remedies to treat many diseases, including cough, colds, asthma, fatigue, gastritis, and respiratory tract infections. Furthermore, its current applications extend to the cosmetic and food industries, such as functional foods and food supplements, owing to its numerous positive effects [2]. Various pharmacological properties of licorice have been reported to treat viral infections, inflammation, oxidant stress, tumors, asthma, diabetes, depression, allergic responses, and menopausal symptoms [3,4,5,6,7]. Therefore, licorice is listed as a medicinal plant in Korean Pharmacopoeia. Three distinct species of licorice among 22 accepted licorice species are listed in the Korean Pharmacopoeia: *G. uralensis* Fisch., *G. glabra* L., and *G. inflata* Batal. Although licorice is one of the most widely-consumed herbal medicines, with 9000–10,000 tons being consumed annually in South Korea, the domestic self-sufficiency rate is 3–5%, due to low productivity, early leaf fall, and insufficient levels of primary components, such as glycyrrhizin and liquiritigenin, as prescribed in the Korean Pharmacopoeia standards (for glycyrrhizin, a minimum of 2.5%, and for liquiritigenin a minimum of 0.7%) [8]. To overcome these problems, a *Glycyrrhiza* new variety, known as Wongam (WG), has been developed by the Korea Rural Development Administration, which is a hybrid of *G. glabra* × *G. uralensis*. WG has been reported as having higher average yields (227%) and higher resistance to brown spot disease and lodging than *G. uralensis*, as well as higher levels of glycyrrhizin (3.96%) and liquiritigenin (0.8%), in compliance with Korean Pharmacopoeia standards. In addition, WG has been shown as having lower cytotoxicity than *G. uralensis* in vitro, while its effects variously include enhancing immune response, as well as anti-allergic and anti-neuroinflammatory properties [9,10,11]. Although several studies have reported the homogeneity and pharmacological effects of WG to register a new species of *Glycyrrhiza* in Korean Pharmacopoeia, a general toxicity study of WG in rodents has not been conducted to date. Therefore, we evaluated the general toxicity of WG in this study.

The general toxicity test is the most basic and fundamental toxicity test, and is used to identify, evaluate, and determine the safety and the possible risk of adverse effects of substances, such as medicines, cosmetics, health foods, chemicals, and pesticides. It is commonly carried out on animals, such as rats, mice, and dogs, because of scientific, ethical, and regulatory reasons [12,13]. The general toxicity test consists of both a single dose and a repeated dose toxicity test, which provide basic toxicity information, including approximate lethal dose (ALD), maximum tolerated dose (MTD), and no-observed-adverse-effect level (NOAEL). The single dose toxicity test provides the most basic information about substances through general symptoms, weight changes, and necropsy findings. The repeated dose toxicity test provides information regarding the long-term effects of substances through general symptoms, weight change, food and water consumption, ophthalmological examination, urinalysis, hematological and blood biochemical tests, necropsy findings, and histopathological examination. Information from the general toxicity test in animals can be used to identify target organ toxicity, to characterize the relationship between drug exposure and response, to determine the likelihood of recovery from drug effects after administration is ended, and to provide information necessary for risk assessment in humans [14,15].

Accordingly, we evaluated the general toxicity of the *Glycyrrhiza* new variety through a single oral dose and a repeated oral dose toxicity test.

## 2. Results

### 2.1. Single Oral Dose Toxicity Study

Over a 15-day observation period, no mortality was observed in rats of either sex at any of the doses tested. Also, there were no abnormal bodyweight changes in either sex at any doses during the study period (Table 1).

No clinical signs related to the administration of WG extract were observed other than compound-colored stool in both sexes of the 2500 mg/kg group on Day 2, and in the 5000 mg/kg on Days 1–2 (Table 2).

In addition, no gross pathological findings were observed at necropsy in rats of either sex treated with WG extract (Table 3).

### 2.2. 13-Week Repeated Oral Dose Toxicity Study

#### 2.2.1. Mortality, Clinical Signs and Ophthalmological Examination

No treatment-related mortality was observed in rats of either sex at any doses during the study period. The compound-colored stool was observed in both sexes of the 1250 mg/kg/day group on Days 7–91, and in the higher dose groups on Days 2–91. Salivation was observed in eight male rats of the 5000 mg/kg/day group on Days 85–91. Loss of fur and scratched wounds were observed in two male rats of the 5000 mg/kg/day on Days 44–92 and Days 16–86, respectively. Crust formation was observed in one male rat of the 5000 mg/kg/day group on Days 17–43. In addition, no treatment-related ophthalmologic abnormalities were observed in rats of either sex at any doses during the study period (Table 4).

#### 2.2.2. Bodyweight and Food and Water Consumption

**Figure 1:**
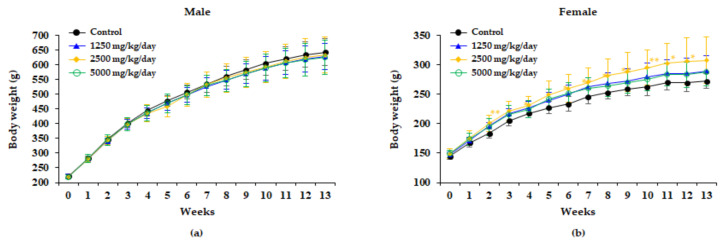
Bodyweight of male (**a**) and female (**b**) rats treated with 0, 1250, 2500, and 5000 mg/kg/day in the 13 weeks repeated oral dose toxicity study of WG extract. The results are presented as the mean ± standard deviation (n = 10). Significantly different from the control group at * *P* < 0.05, ** *P* < 0.01.

No significant differences in bodyweight between the control and treatment groups were observed in male rats during the study period. However, a significant increase in bodyweight was observed in female rats of the 2500 mg/kg/day group in weeks 2, 3, 5, 6, 7, 8, 10, 11, and 12 (Figure 1).

**Figure 2:**
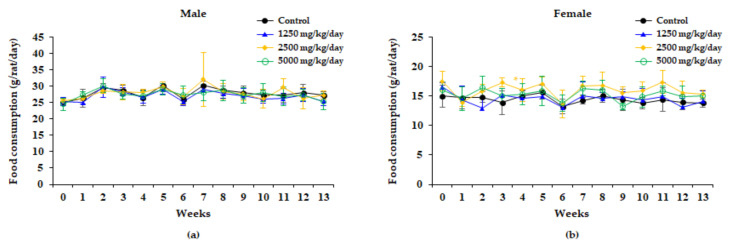
Food consumption of male (**a**) and female (**b**) rats treated with 0, 1250, 2500, and 5000 mg/kg/day in the 13 weeks repeated oral dose toxicity study of WG extract. Results are presented as the mean ± standard deviation (n = 10). Significantly different from the control group at * *P* < 0.05.

Food consumption in male rats was not significantly different between the control and treatment groups during the study period. In female rats of the 2500 mg/kg/day group, however, food consumption increased significantly on Day 21 compared to the control group (Figure 2).

**Figure 3:**
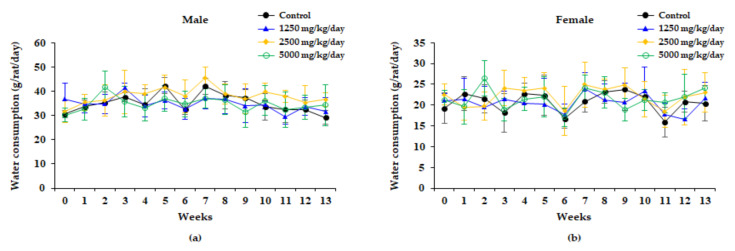
Water consumption of male (**a**) and female (**b**) rats treated with 0, 1250, 2500, and 5000 mg/kg/day in the 13 weeks repeated oral dose toxicity study of WG extract. Results are presented as the mean ± standard deviation (n = 10).

There were no significant changes in water consumption between the control and treatment groups in rats of either sex at any doses during the study period (Figure 3).

#### 2.2.3. Urinalysis

The results of urinalysis are shown in Table 5 (male rats) and Table 6 (female rats). Ketone body was increased in both sexes of the 5000 mg/kg/day group. Also, specific gravity was increased in male rats of the 2500 and 5000 mg/kg/day groups.

#### 2.2.4. Hematology

Table 7 and Table 8 summarize the results of hematological tests involving male and female rats, respectively. No significant differences were observed in any parameters between the control and either of the dose treatment groups rats of either sex.

#### 2.2.5. Serum Biochemistry

The results of serum biochemistry are presented in Table 9 (male rats) and Table 10 (female rats). In male rats, Na was significantly increased in the 2500 and 5000 mg/kg/day groups. In female rats, ALP was significantly increased in the 5000 mg/kg/day group.

#### 2.2.6. Absolute and Relative Organ Weight

The results of absolute and relative organ weight in male and female rats are shown in Table 11 and Table 12, respectively. In male rats, the relative organ weight of the left kidney in the 5000 mg/kg/day group and the absolute organ weight of the right testis in the 1250 mg/kg/day group were significantly increased. In female rats, the absolute organ weight of the lung was increased in the 1250 and 2500 mg/kg/day groups. Additionally, bodyweights were significantly increased in female rats of the 2500 mg/kg/day group.

#### 2.2.7. Necropsy Findings

Table 13 and Table 14 summarize the results of necropsy findings in male and female rats, respectively. In one male rat of the control group, the absence of the left parathyroid gland and the left thyroid gland, and enlargement of the right thyroid gland were observed. In one male rat of the 5000 mg/kg/day group, alopecia and skin crusting were observed. Retention of clear fluid in the uterus was observed in one female rat in the control group, three female rats of the 1250 mg/kg/day group, two female rats of the 2500 mg/kg/day group, and one female rat of the 5000 mg/kg/day group. In addition, discoloration of the liver was observed in one female rat of the 1250 mg/kg/day group.

#### 2.2.8. Histopathology

Histopathological examination was conducted in the control group and the highest dose group (5000 mg/kg/day), and organs with abnormal findings the identified in necropsy examination (data not shown). Luminal dilation of the uterus with retention of clear fluid was observed in one female rat in the control group, three female rats of the 1250 mg/kg/day group, two female of the 2500 mg/kg/day group, and one female rat of the 5000 mg/kg/day group, respectively. In addition, necrosis lesions in the liver with discoloration were observed in one female rat of the 1250 mg/kg/day group.

## 3. Discussion

In this study, the potential toxicity of the WG extract was evaluated by a single oral dose toxicity study, and by 4-week and 13-week repeated oral dose toxicity studies using Sprague-Dawley rats. The information about the safety of the WG extract obtained through the general toxicity study, including gross toxicity, NOAEL, and target organs, will be contributed to registering the *Glycyrrhiza* new variety (Wongam) in the Korean Pharmacopoeia of the Ministry of Food and Drug Safety.

In the single oral dose toxicity study, oral administration of WG extract did not cause mortality or acute toxicity in rats, and we suggest that the ALD of WG extract is higher than 5000 mg/kg. Compound-colored stool was observed in both sexes of the 2500 and 5000 mg/kg groups, which was assumed to result from tinting of the stool, due to the color of WG or its excreted metabolites. However, this finding was not considered to be an adverse effect, since it occurred only transiently and ultimately disappeared, with no other abnormal findings in bodyweight or gross pathology at necropsy (Table 1, Table 2 and Table 3).

In the 4-week repeated oral dose toxicity study, no treatment-related adverse effects were observed involving mortality, clinical signs, ophthalmological examination, bodyweight, food and water consumption, urinalysis, hematology, serum biochemistry, absolute and relative organ weights, or necropsy, at doses up to 5000 mg/kg/day. No deaths or ophthalmologic abnormalities were observed in either sex (Table S1). As in the single oral dose toxicity study, compound-colored stool was observed in both sexes of the 1250 mg/kg/day and higher dose groups, attributable to the color of WG or its excreted metabolites. Although the loss of fur was observed in one male rat in the 1250 mg/kg/day group, this was not considered as related to administering the WG extract owing to the very low incidence rate and the absence of a clear relationship between dose and response. Moreover, loss of fur occurring spontaneously following systemic administration has been reported in previous toxicity studies [16]. Salivation was observed in three male rats of the 5000 mg/kg/day group, which was considered as related to administering the WG extract because it occurred in the highest dose group. However, it was considered to be a temporary physiological reaction resulting from features of WG extract, such as taste and smell.

Bodyweight was not significantly changed in the treatment groups compared to the control group during the 4-week repeated oral dose toxicity study (Figure S1). Although food consumption significantly increased in the 625 and 5000 mg/kg/day groups, and significantly decreased in female rats of the 2500 and 5000 mg/kg/day groups, this was not attributed to administering the WG extract, since it occurred transiently and was not accompanied by any corresponding weight changes (Figure S2). Similarly, the significantly increased water consumption of female rats in all dose groups, and the significantly decreased water consumption of male rats of the 625 and 1250 mg/kg/day groups and female rats of the 625, 1250, and 2500 mg/kg/day groups were not considered to be related to the WG extract, since the responses were sporadic and not dose-related, with no corresponding changes in bodyweight or serum biochemistry (Figure S3). Furthermore, these changes in food and water consumption remained within the normal range of the reference data [17].

During the 4-week repeated oral dose toxicity study, no significant changes were observed in urinalysis (Tables S2 and S3), hematology (Tables S4 and S5), serum biochemistry (Tables S6 and S7), or organ weights (Tables S8 and S9) in any of the WG-extract dose groups and in rats of either sex compared to the control group.

Hydronephrosis in the right kidney of one male rat treated with 625 mg/kg/day group was found at necropsy (Tables S10 and S11) during the 4-week repeated oral dose toxicity study. However, this was not considered to be toxicologically significant owing to the very low incidence rate and the absence of any correlation with dose-dependent responses. Moreover, the response was not attributable to the WG extract in this study, since it has been reported as occurring spontaneously in Sprague-Dawley rats in previous studies [18,19]. A histopathological examination was not performed, as there were no organs with significant gross lesions.

In the 13-week repeated oral dose toxicity study, no death or ophthalmologic abnormalities were observed in rats of either sex at doses up to 5000 mg/kg/day. Similarly to the single oral dose toxicity study and the 4-week repeated oral dose toxicity study, compound-colored stool and salivation were observed in all dose groups in both sexes and in eight male rats of the 5000 mg/kg/day group, respectively. Loss of fur and wound scratching were observed in two male rats of the 5000 mg/kg/day group, and crusted skin formation was observed in one of these (Table 4). These signs were not considered as related to administering the WG extract for the same reasons as with the 4-week repeated oral dose toxicity study.

No significant changes in bodyweight or food consumption were observed in male rats in relation to administering the WG extracts. By contrast, bodyweight was significantly increased during most experiment periods involving female rats of the 2500 mg/kg/day group, while food consumption was significantly increased at week 3 in female rats of the 2500 mg/kg/day group. These increases were not considered as related to administering the WG extract, since they were not dose-related (Figure 1 and Figure 2). Water consumption did not significantly change after administration WG extract in any of the dose groups of either sex compared to the control group (Figure 3).

In the urinalysis tests, the ketone body was increased in both sexes of the 5000 mg/kg/day group. Specific gravity was increased in male rats of the 2500 and 5000 mg/kg/day groups (Table 5 and Table 6). These changes were related to administering the WG extract because the responses were dose-dependent and accompanied by increasing kidney weights. However, they were not considered to be toxicologically significant, since they remained within the normal range of the reference data and were not accompanied by histopathological changes in other related items [17].

In the hematology tests, RET tended to decrease in male rats of the 2500 and 5000 mg/kg/day group (Table 7). However, this was not considered as related to administering the WG extracts, since the RET ratio of the control group in this study was higher than the normal levels in other reports with the same conditions [20,21,22,23,24].

In the serum biochemistry tests, Na was significantly increased in male rats of the 2500 and 5000 mg/kg/day group (Table 9). ALP was significantly increased in female rats of the 5000 mg/kg/day group (Table 10). These changes were considered as related to administering the WG extract, since the responses were dose-dependent. However, they were not considered to be toxicologically significant, since they remained within the normal range of the reference data and were not accompanied by histopathological changes in other related items [17].

The relative organ weight of the left kidney and the absolute organ weight of the right testis were significantly increased in male rats of the 5000 mg/kg/day and 1250 mg/kg/day groups, respectively (Table 11). In addition, the absolute organ weight of the lung was significantly increased in female rats of the 1250 and 2500 mg/kg/day groups (Table 12). These changes were not considered as related to administering the WG extract, since the responses were not dose-related.

In the necropsy findings (Table 13 and Table 14) and histopathological tests (data not shown), the absence of the left parathyroid gland and the left thyroid gland, and enlargement of the right thyroid gland were observed in one male rat in the control group. Alopecia and skin crusting were observed in one male rat of the 5000 mg/kg/day group through necropsy and histopathology tests. Retention of clear fluid in the uterus in the necropsy findings was caused by luminal dilation, observed in histopathological tests. Discoloration of the liver, observed in necropsy findings, was caused by focal/multifocal necrosis, which was also observed in histopathological tests. These changes were judged to be neither significant nor dose-related, having occurred spontaneously in Sprague-Dawley rats [25]. Furthermore, necropsy findings did not correlate with histopathology.

Interestingly, chronic ingestion of licorice or licorice-like compounds (such as carbenoxolone) could lead to the temporary form of apparent mineralocorticoid excess (AME), which is an autosomal recessive disorder. AME is characterized by hypertension, hypokalemia, metabolic alkalosis, and low plasma renin activity, and aldosterone level [26]. The major etiology of AME is mutations in the 11β-hydroxysteroid dehydrogenase type 2 (11β-HSD2) gene, which catalyzes the conversion of active cortisol to inactive cortisol in the kidney. When a 11β-HSD2 mutation occurrs, it causes high concentrations of cortisol in the kidney. Then it can cross-react and activate the mineralocorticoid receptor, which leads to the AME-associated syndromes [27,28]. It is noteworthy that glycyrrhetinic acid, which is the hydrolysis of glycyrrhizic acid, has been identified as an inhibitor of 11β-HSD2 [26]. Therefore, long term ingestion of licorice could occur the possibility of AME. In our results, there are no abnormal signs associated with AME in the single oral dose and 4-week repeated oral dose toxicity study. However, a significantly increase of the urinary ketone body and protein (Table 5 and Table 6), serum Na (Table 9), and kidney weight (Table 11) occurred in a 13-week repeated oral dose toxicity study. Although we considered that these changes were not toxicologically significant, due to the within the normal range of the reference data and not accompanied by histopathological changes, it seemed to be a possibility of AME development. Besides, WG has higher levels of glycyrrhizic acid than other species of licorice. Additionally, previous studies reported that AME-associated symptoms are not present with consumption of less than 50 g/day of licorice, although several practical difficulties [29]. Human equivalent dose (HED) upon the 5000 mg/kg of WG extracts in rats is almost 811 mg/kg [30]. Therefore, we suggest that long term treatment of WG high dose should be carefully given to patients, due to the possibility of AME.

Toxicological effects of the *Glycyrrhiza* radix have been reported in many previous studies. In a single oral dose toxicity study of *G. glabra* in rats, ethanol and aqueous extracts did not cause mortality at up to 1000 mg/kg over 14 days. In the administration of the 2000 mg/kg of these extracts, slight gross behavioral changes were observed, including alertness, spontaneous locomotor activity, and reactivity to touch [31]. A 15-day repeated oral dose toxicity of *G. glabra*, aqueous extract (100, 250, and 500 mg/kg/day) in rats is reported in one study. Suppression of the adrenal-pituitary axis, decreased plasma concentrations of cortisol, adrenocorticotropic hormone (ACTH), aldosterone, and K+, and increased plasma concentrations of renin and Na+ were observed in a dose-dependent manner [32]. In addition, a 9-week repeated oral dose toxicity study of water extract of *G. uralensis* was conducted in rats. The NOAEL of water extract of *G. uralensis* was reported to be higher than 2000 mg/kg/day in male rats, despite a slight decrease in prostate weight and daily sperm production [33]. A 13-week repeated oral dose toxicity study was conducted with flavonoid oil of *G. glabra* instead of water extract. The NOAEL of flavonoid oil of *G. glabra* was estimated to be 400 mg/kg/day in male rats, and 800 mg/kg/day in female rats [34]. Although we only estimated the general toxicity of WG in this study, and not the toxicity of other *Glycyrrhiza* species, we could infer that the toxicity of other *Glycyrrhiza* species is higher than that of the *Glycyrrhiza* new variety (Wongam) through comparison with the previous reports.

In conclusion, we report that the oral ALD and NOAEL of WG are higher than 5000 mg/kg in rats of both sexes, and that no target organs were identified. We accordingly assume that WG is of lower general toxicity than that of other *Glycyrrhiza* species reported in previous studies. The results of the present study are thus expected to contribute to the registration of the *Glycyrrhiza* new variety (Wongam) in the Korean Pharmacopoeia.

## 4. Materials and Methods

### 4.1. Wongam Preparation

WG was obtained from the Korean Rural Development Administration, and extracted by Wonkwang Herb Co. (Jinan-gun, Jeollabuk-do, Republic of Korea). Briefly, WG was extracted with distilled water at 100 ℃ for 4 h 30 min. The extract was concentrated under reduced pressure in a rotary evaporator at 70 ℃ for 3 h. The decoction was filtered using Whatman filter paper no.1. Then, it was lyophilized (Batch methods) and stored at 4 ℃. The yield of the dried extract from the starting crude of the WG was 8.8%. The WG extract was prepared for administration by suspending with sterile water for injection according to the doses assigned for each group in the single and repeated oral dose toxicity studies.

### 4.2. Experimental Animals and Animal Husbandry

All toxicological studies were carried out by Chemon Inc. under Good Laboratory Practice (GLP) conditions. Specific pathogen-free Sprague-Dawley rats were obtained from Orient bio Inc. (Gapyeong, Gyeonggi-do, Republic of Korea), and used for the single and repeated oral dose toxicity studies. The studies were approved by the Institutional Animal Care and Use Committee (IACUC) of the Preclinical Research Center, Chemon Inc. (Approval Number: 19-R491 for the single oral dose toxicity study and 19-R654 for the 13-week repeated oral dose toxicity study). Animals were housed in the laboratory animal facility at a temperature of 23 ± 3°C and relative humidity of 55 ± 15%. Animal housing was maintained under a 12-h light-dark cycle, with 10–20 air changes per hour. Animals were supplied irradiation-sterilized pellet feed (Teklad Certified Irradiated Global 18% Protein Rodent Diet, 2918C; Envigo RMS, Inc., IN, USA.), along with tap water disinfected using an ultraviolet sterilizer and ultrafiltration ad libitum. All animals were acclimated for 6 days before the start of the experiment. The study was conducted in accordance with test guidelines from the Korean Ministry of Food and Drug Safety (MFDS, 2018) and guidelines for the testing of chemicals from the Organization for Economic Cooperation and Development (OECD, 1997) under GLP Regulations.

### 4.3. Single Oral Dose Toxicity Study

8-week-old Sprague-Dawley male and female rats (n = 5 per sex and group) were orally treated with WG at 0 (Control), 1250, 2500, and 5000 mg/kg. Animals were housed in stainless steel cages with mesh flooring. No more than three animals were housed per cage during the quarantine and acclimation period, and the animals were housed individually during the dosing and observation periods. Clinical signs and mortality were monitored constantly for the first 30 min after dose administration, then every hour until 6 h after oral treatment, and daily for 14 days subsequently. During the 15-day experimental period, the bodyweight of all rat groups was recorded. Then, all animals were euthanized by inhaled carbon dioxide (CO2) on Day 15, and gross findings were observed at necropsy.

### 4.4. 13-Week Repeated Oral Dose Toxicity Study

The high dose was set at 5000 mg/kg/day, based on the absence of toxic signs in a 4-week repeated oral dose toxicity study (Figures S1–S3 and Tables S1–S11). 6-week-old male and female Sprague-Dawley rats (n = 10 per sex and group) were orally administered WG at 0 (control), 1250, 2500, and 5000 mg/kg/day for 13 weeks. Bodyweight ranges at the initiation of dosing were 207.93–235.53 g for males and 136.04–165.50 g for females, respectively.

Animals were individually checked once a day to observe any clinical signs and mortalities, and the type, date of occurrence, and severity of signs were recorded. Bodyweights of all rat groups were recorded before the initiation of dosing (Day 1) and once a week during the experimental period. Before necropsy, all rat groups were fasted overnight, and bodyweights were recorded at necropsy. Food and water intake were checked on the same days as bodyweight measurements were recorded. The eyes of five male and five female rats per group were macroscopically evaluated during the final week of observation. A mydriatic (Mydriacyl Eye Drops 1%, Alcon Korea) was dropped into both eyeballs to facilitate mydriasis, after which the anterior parts of the eye, optic media, and fundus were observed with a Keeler Vantage Plus LED Digital Binocular Ophthalmoscope (Keeler Instruments Inc., PA, USA).

Urinalysis was performed during the last week of observation; 5 male and 5 female rats per group were individually housed in a stainless-steel cage cleaned and disinfected with 70% alcohol. Urine samples were collected, and 0.3 mL of fresh urine was taken for analysis. Urine samples were analyzed for glucose, bilirubin, ketone body, specific gravity, pH, protein, urobilinogen, nitrite, occult blood, red blood cell (RBC), white blood cell (WBC), epithelial cell, urine color, clarity, cast, and total urine volume using an automatic analyzer (Clinitek Advantus; Siemens, Munchen, Germany).

For necropsy, animals were euthanized by inhalation of 3–5% isoflurane (Terel liquid; Kyongbo Pharma. Co., Ltd., Asan-si, Chungcheongnam-do, Republic of Korea) on Day 91. Blood samples were collected from the posterior vena cava for hematological and serum biochemical testing. Approximately 1 mL of blood was placed in a CBC bottle (Vacutainer 3 mL; BD) with anticoagulant EDTA-2K. Hematology parameters were measured using a hematology analyzer (ADVIA 2120; Siemens, Munchen, Germany), including RBC, hemoglobin (HGB), hematocrit (HCT), mean corpuscular volume (MCV), mean corpuscular hemoglobin (MCH), mean corpuscular hemoglobin concentration (MCHC), red cell distribution width (RDW), hemoglobin distribution width (HDW), reticulocyte (RET), platelet count (PLT), mean platelet volume (MPV), white blood cell count (WBC), neutrophils count (NEU), lymphocytes count (LYM), monocytes count (MONO), eosinophils count (EOS), basophils count (BASO), and large unstained cell count (LUC). Activated partial thromboplastin time (APTT) and prothrombin time (PT) were measured using a coagulation analyzer (CS-1600; Sysmex; Kobe, Hyogo, Japan).

Serum biochemical parameters were measured using a serum biochemistry analyzer (AU680; Beckman Coulter; CA, USA). About 2 mL of the blood sample was added to a 5 mL Vacutainer tube (SST™ II Advance; BD; NJ, USA) that contained a clot activator. Blood was coagulated by being left at room temperature for 15–20 min and then centrifuged for 10 min (3000 rpm, 1902 Relative Centrifugal Force (RCF), Combi-514R; Hanil, Republic of Korea) to collect serum samples. Parameters examined were aspartate aminotransferase (AST), alanine aminotransferase (ALT), alkaline phosphatase (ALP), creatine phosphokinase (CPK), total bilirubin (TBIL), glucose (GLU), total cholesterol (TCHO), triglyceride (TG), total protein (TP), albumin (ALB), albumin/globulin (A/G) ratio, blood urea nitrogen (BUN), creatinine (CREA), inorganic phosphorus (IP), calcium (Ca), sodium (Na), potassium ion (K) and chloride ion (Cl).

After blood sampling, animals were sacrificed by exsanguination from the abdominal aorta and posterior vena cava. Gross findings were recorded, including body surface, subcutis, head, and all organs in the abdominal and thoracic cavities. Following this, organs were weighed using an electronic balance (Sartorius AG): brain, pituitary gland, lung heart, thymus, spleen, adrenal (both), kidney (both), liver, testis (both), epididymis (both), and prostate gland. Organ weights (%) relative to terminal bodyweights were also calculated.

Histopathological examination was performed on all organs collected from the control and highest dose (5000 mg/kg/day) groups, and additionally on macroscopically abnormal organs from the low- and mid-dose groups.

### 4.5. Statistical Analysis

Data are presented as mean ± standard deviation. Statistical analysis was performed by parametric one-way analysis of variance (ANOVA), assumption of homogeneity was performed by Levene’s test, and evaluation of significant differences between the vehicle control and treatment groups was estimated by Dunnett’s test using the Provantis^TM^ 10.10.1 package. The level of significance was taken as *P* < 0.05.

In this study, unless specified otherwise, the term “significant” in sentences with P-value implies that inter-group differences have attained statistical significance compared to the control group.

## Figures and Tables

**Table 1:** Bodyweight and mortality in single oral dose toxicity of WG extract.

Sex	Dose (mg/kg)	Bodyweight (g) (Mean ± SD)					Mortality (Dead/Total)
		Day 1	Day 2	Day 4	Day 8	Day 15	
Male	Control	281.466 ± 6.114	314.398 ± 7.703	331.808 ± 12.329	362.900 ± 11.915	410.050 ± 20.768	0% (0/5)
	1250	280.804 ± 7.599	311.880 ± 9.621	329.594 ± 5.731	363.068 ± 12.778	417.776 ± 19.317	0% (0/5)
	2500	276.854 ± 6.174	310.744 ± 7.577	327.868 ± 10.002	358.008 ± 14.395	410.658 ± 27.829	0% (0/5)
	5000	279.206 ± 8.008	312.442 ± 8.767	330.470 ± 11.954	362.086 ± 20.912	409.972 ± 38.043	0% (0/5)
Female	Control	197.842 ± 6.051	220.122 ± 8.425	227.518 ± 10.747	234.928 ± 12.446	257.224 ± 29.331	0% (0/5)
	1250	198.494 ± 7.890	217.092 ± 13.885	227.740 ± 10.210	237.494 ± 11.940	252.028 ± 17.321	0% (0/5)
	2500	199.578 ± 4.264	220.060 ± 7.402	226.420 ± 7.891	235.222 ± 7.385	247.224 ± 12.209	0% (0/5)
	5000	196.380 ± 7.204	214.182 ± 7.826	225.738 ± 10.114	238.622 ± 11.151	248.528 ± 14.090	0% (0/5)

Significant differences were compared with the control group.

**Table 2:** Clinical signs in single oral dose toxicity of WG extract.

Sex	Dose (mg/kg)	Observation Type	Days Relative to Start Date																			
			1	1	1	1	1	1	2	3	4	5	6	7	8	9	10	11	12	13	14	15
			1 hr	2 hr	3 hr	4 hr	5 hr	6 hr														
Male	Control	Normal	5	5	5	5	5	5	5	5	5	5	5	5	5	5	5	5	5	5	5	5
	1250	Normal	5	5	5	5	5	5	5	5	5	5	5	5	5	5	5	5	5	5	5	5
	2500	Normal	5	5	5	5	5	5	0	5	5	5	5	5	5	5	5	5	5	5	5	5
		Compound-colored stool	0	0	0	0	0	0	5	0	0	0	0	0	0	0	0	0	0	0	0	0
	5000	Normal	5	5	5	5	0	0	0	5	5	5	5	5	5	5	5	5	5	5	5	5
		Compound-colored stool	0	0	0	0	5	5	5	0	0	0	0	0	0	0	0	0	0	0	0	0
No. of animals			5	5	5	5	5	5	5	5	5	5	5	5	5	5	5	5	5	5	5	5
Female	Control	Normal	5	5	5	5	5	5	5	5	5	5	5	5	5	5	5	5	5	5	5	5
	1250	Normal	5	5	5	5	5	5	5	5	5	5	5	5	5	5	5	5	5	5	5	5
	2500	Normal	5	5	5	5	5	5	0	5	5	5	5	5	5	5	5	5	5	5	5	5
		Compound-colored stool	0	0	0	0	0	0	5	0	0	0	0	0	0	0	0	0	0	0	0	0
	5000	Normal	5	5	5	5	5	0	0	5	5	5	5	5	5	5	5	5	5	5	5	5
		Compound-colored stool	0	0	0	0	0	5	5	0	0	0	0	0	0	0	0	0	0	0	0	0
No. of animals			5	5	5	5	5	5	5	5	5	5	5	5	5	5	5	5	5	5	5	5

**Table 3:** Necropsy findings in single oral dose toxicity of WG extract.

Parameters	Observation Type	Male				Female			
		Dose (mg/kg)				Dose (mg/kg)			
		Control	1250	2500	5000	Control	1250	2500	5000
Adrenal Gland	Normal	5	5	5	5	5	5	5	5
Aorta	Normal	5	5	5	5	5	5	5	5
Bone Marrow, Sternum	Normal	5	5	5	5	5	5	5	5
Brain	Normal	5	5	5	5	5	5	5	5
Cervix	Normal	-	-	-	-	5	5	5	5
Coagulating Gland	Normal	5	5	5	5	-	-	-	-
Epididymis	Normal	5	5	5	5	-	-	-	-
Esophagus	Normal	5	5	5	5	5	5	5	5
Eye with Optic Nerve	Normal	5	5	5	5	5	5	5	5
Harderian Gland	Normal	5	5	5	5	5	5	5	5
Heart	Normal	5	5	5	5	5	5	5	5
Cecum	Normal	5	5	5	5	5	5	5	5
Colon	Normal	5	5	5	5	5	5	5	5
Duodenum	Normal	5	5	5	5	5	5	5	5
Ileum	Normal	5	5	5	5	5	5	5	5
Jejunum	Normal	5	5	5	5	5	5	5	5
Rectum	Normal	5	5	5	5	5	5	5	5
Kidney	Normal	5	5	5	5	5	5	5	5
Liver	Normal	5	5	5	5	5	5	5	5
Lung	Normal	5	5	5	5	5	5	5	5
Lymph Node, Mesenteric	Normal	5	5	5	5	5	5	5	5
Lymph Node, Mandibular	Normal	5	5	5	5	5	5	5	5
Skeletal Muscle	Normal	5	5	5	5	5	5	5	5
Nerve, Peripheral	Normal	5	5	5	5	5	5	5	5
Ovary	Normal	-	-	-	-	5	5	5	5
Pancreas	Normal	5	5	5	5	5	5	5	5
Parathyroid Gland	Normal	5	5	5	5	5	5	5	5
Pituitary Gland	Normal	5	5	5	5	5	5	5	5
Prostate Gland	Normal	5	5	5	5	-	-	-	-
Salivary Gland	Normal	5	5	5	5	5	5	5	5
Seminal Vesicle	Normal	5	5	5	5	-	-	-	-
Skin, Mammary	Normal	5	5	5	5	5	5	5	5
Spinal Cord, Thoracic	Normal	5	5	5	5	5	5	5	5
Spleen	Normal	5	5	5	5	5	5	5	5
Stomach	Normal	5	5	5	5	5	5	5	5
Testis	Normal	5	5	5	5	-	-	-	-
Thymus	Normal	5	5	5	5	5	5	5	5
Thyroid Gland	Normal	5	5	5	5	5	5	5	5
Tongue	Normal	5	5	5	5	5	5	5	5
Trachea	Normal	5	5	5	5	5	5	5	5
Urinary Bladder	Normal	5	5	5	5	5	5	5	5
Uterus	Normal	-	-	-	-	5	5	5	5
Vagina	Normal	-	-	-	-	5	5	5	5
Femorotibial Joint	Normal	5	5	5	5	5	5	5	5
No. of animals		5	5	5	5	5	5	5	5

-: Not applicable.

**Table 4:** Mortality, clinical signs, and ophthalmological examination in 13 weeks repeated oral dose toxicity of WG extract.

Observation Type	From Week 0 to Week 13							
	Male				Female			
	Dose (mg/kg/day)				Dose (mg/kg/day)			
	Control	1250	2500	5000	Control	1250	2500	5000
Normal	10	0	0	0	10	0	0	0
Compound-colored stool	0	10	10	10	0	10	10	10
Salivation	0	0	0	8	0	0	0	0
Loss of fur	0	0	0	2	0	0	0	0
Scratched wound	0	0	0	2	0	0	0	0
Crust formation	0	0	0	1	0	0	0	0
Mortality (dead/total)	0% (0/10)	0% (0/10)	0% (0/10)	0% (0/10)	0% (0/10)	0% (0/10)	0% (0/10)	0% (0/10)
No. of animals	10	10	10	10	10	10	10	10
Ophthalmological examination	N	N	N	N	N	N	N	N
No. of animals	5	5	5	5	5	5	5	5

N, Normal.

**Table 5:** Urinalysis in 13 weeks repeated oral dose toxicity of WG extract in male rats.

Parameters	Result	Dose (mg/kg/day)			
		Control	1250	2500	5000
Male					
Glucose	-	5	5	5	5
Bilirubin	-	5	5	5	5
Ketone body	-	5	5	4	1
	+/−	0	0	0	0
	1+	0	0	1	3
	2+	0	0	0	1
	3+	0	0	0	0
Specific gravity	≤1.005	0	0	0	0
	1.010	5	4	4	1
	1.015	0	1	1	2
	1.020	0	0	0	2
pH	7.5	0	0	0	0
	8.0	2	4	4	0
	8.5	3	1	1	5
	≥9.0	0	0	0	0
Protein	-	5	3	4	1
	+/−	0	2	1	2
	1+	0	0	0	2
	2+	0	0	0	0
	3+	0	0	0	0
Urobilinogen	0.2	5	5	5	5
	1.0	0	0	0	0
Nitrite	-	5	5	5	5
	+	0	0	0	0
Occult blood	-	3	4	4	5
	+/−	2	1	1	0
	1+	0	0	0	0
	2+	0	0	0	0
	3+	0	0	0	0
Clarity		5	5	5	5
Color	Yellow	5	5	5	5
Volume ^(a)^	mL	18.80 ± 8.70	16.00 ± 3.00	18.60 ± 3.51	15.00 ± 4.64
RBC	-	4	5	4	5
	+/−	1	0	1	0
	1+	0	0	0	0
	2+	0	0	0	0
WBC	-	5	5	5	5
	+/−	0	0	0	0
	1+	0	0	0	0
	2+	0	0	0	0
	3+	0	0	0	0
Epithelial cell	-	5	5	5	5
	+/−	0	0	0	0
	1+	0	0	0	0
Casts	-	5	5	5	5
	+/−	0	0	0	0
	1+	0	0	0	0
No. of animals		5	5	5	5

RBC, red blood cell; WBC, white blood cell. ^(a)^ Values are mean ± standard deviation. Significant differences were compared with the control group.

**Table 6:** Urinalysis in 13 weeks repeated oral dose toxicity of WG extract in female rats.

Parameters	Result	Dose (mg/kg/day)			
		Control	1250	2500	5000
Female					
Glucose	-	5	5	5	5
Bilirubin	-	5	5	5	5
Ketone body	-	5	5	5	0
	+/−	0	0	0	2
	1+	0	0	0	3
	2+	0	0	0	0
	3+	0	0	0	0
Specific gravity	≤1.005	0	0	0	0
	1.010	2	5	4	2
	1.015	3	0	1	3
	1.020	0	0	0	0
pH	7.5	0	0	1	0
	8.0	0	1	0	1
	8.5	5	4	4	4
	≥9.0	0	0	0	0
Protein	-	5	5	5	4
	+/−	0	0	0	1
	1+	0	0	0	0
	2+	0	0	0	0
	3+	0	0	0	0
Urobilinogen	0.2	5	5	5	5
	1.0	0	0	0	0
Nitrite	-	5	5	5	5
	+	0	0	0	0
Occult blood	-	5	5	5	5
	+/−	0	0	0	0
	1+	0	0	0	0
	2+	0	0	0	0
	3+	0	0	0	0
Clarity		5	5	5	5
Color	Yellow	5	5	5	5
Volume ^(a)^	mL	10.40 ± 1.67	11.80 ± 4.44	12.60 ± 3.85	9.60 ± 2.30
RBC	-	5	5	5	5
	+/−	0	0	0	0
	1+	0	0	0	0
	2+	0	0	0	0
WBC	-	5	5	5	5
	+/−	0	0	0	0
	1+	0	0	0	0
	2+	0	0	0	0
	3+	0	0	0	0
Epithelial cell	-	5	5	5	5
	+/−	0	0	0	0
	1+	0	0	0	0
Casts	-	5	5	5	5
	+/−	0	0	0	0
	1+	0	0	0	0
No. of animals		5	5	5	5

RBC, red blood cell; WBC, white blood cell. ^(a)^ Values are mean ± standard deviation. Significant differences were compared with the control group.

**Table 7:** Hematology values in 13 weeks repeated oral dose toxicity of WG extract in male rats.

Parameters	Dose (mg/kg/day)			
	Control	1250	2500	5000
Male				
RBC (10^6^/µL)	8.361 ± 0.390	8.323 ± 0.278	8.341 ± 0.401	8.562 ± 0.295
HGB (g/dL)	14.940 ± 0.540	15.03 ± 0.67	15.21 ± 0.66	15.20 ± 0.68
HCT (%)	44.93 ± 1.31	45.19 ± 1.88	45.60 ± 1.94	45.55 ± 2.06
MCV (fL)	53.82 ± 1.91	54.31 ± 1.78	54.74 ± 0.95	53.22 ± 1.25
MCH (pg)	17.87 ± 0.76	18.05 ± 0.53	18.25 ± 0.29	17.77 ± 0.48
MCHC (g/dL)	33.21 ± 0.56	33.24 ± 0.47	33.35 ± 0.35	33.40 ± 0.46
RDW (%)	13.13 ± 0.79	13.23 ± 1.10	13.04 ± 0.68	13.09 ± 0.83
HDW (g/dL)	2.694 ± 0.196	2.740 ± 0.295	2.715 ± 0.276	2.645 ± 0.249
RET (%)	2.936 ± 0.945	2.708 ± 0.623	2.542 ± 0.591	2.392 ± 0.324
PLT (10^3^/µL)	981.7 ± 134.1	1018.1 ± 89.6	1032.1 ± 84.5	1034.8 ± 88.3
MPV (fL)	7.14 ± 0.35	6.97 ± 0.52	6.89 ± 0.47	6.81 ± 0.57
WBC (10^3^/µL)	11.081 ± 2.710	10.770 ± 1.725	11.430 ± 2.801	10.761 ± 2.370
NEU (%)	17.80 ± 4.52	15.84 ± 5.35	17.01 ± 7.46	17.67 ± 3.24
NEU (10^3^/µL)	1.923 ± 0.448	1.705 ± 0.679	1.965 ± 1.133	1.893 ± 0.524
LYM (%)	77.85 ± 4.97	79.52 ± 5.69	77.97 ± 8.03	78.29 ± 3.78
LYM (10^3^/µL)	8.680 ± 2.438	8.565 ± 1.474	8.894 ± 2.245	8.437 ± 1.959
MONO (%)	2.78 ± 0.67	2.77 ± 1.01	2.96 ± 0.98	2.46 ± 0.58
MONO (10^3^/µL)	0.301 ± 0.089	0.295 ± 0.105	0.341 ± 0.151	0.263 ± 0.081
EOS (%)	0.94 ± 0.31	1.15 ± 0.38	1.25 ± 0.41	1.00 ± 0.41
EOS (10^3^/µL)	0.103 ± 0.029	0.122 ± 0.039	0.136 ± 0.044	0.105 ± 0.033
BASO (%)	0.14 ± 0.07	0.15 ± 0.07	0.17 ± 0.07	0.16 ± 0.05
BASO (10^3^/µL)	0.018 ± 0.016	0.017 ± 0.009	0.018 ± 0.011	0.016 ± 0.007
LUC (%)	0.50 ± 0.14	0.60 ± 0.30	0.68 ± 0.36	0.46 ± 0.12
LUC (10^3^/µL)	0.058 ± 0.030	0.065 ± 0.039	0.075 ± 0.039	0.048 ± 0.018
APTT (sec)	15.28 ± 0.62	14.52 ± 1.98	15.76 ± 0.84	15.16 ± 1.05
PT (sec)	8.54 ± 0.21	8.63 ± 0.38	8.80 ± 0.21	8.53 ± 0.22
No. of animals	10	10	10	10

RBC, red blood cell; HGB, hemoglobin; HCT, hematocrit; MCV; mean corpuscular volume; MCH, mean corpuscular hemoglobin; MCHC, mean corpuscular hemoglobin concentration; RDW, red cell distribution width; HDW, hemoglobin distribution width; RET, reticulocyte; PLT, platelet; MPV, mean platelet volume; WBC, white blood cell; NEU, neutrophils; LYM, lymphocytes; MONO, monocytes; EOS, eosinophils; BASO, basophils; LUC, large unstained cell, APTT, activated partial thromboplastin time; PT, prothrombin time. Significant differences were compared with the control group.

**Table 8:** Hematology values in 13 weeks repeated oral dose toxicity of WG extract in female rats.

Parameters	Dose (mg/kg/day)			
	Control	1250	2500	5000
Female				
RBC (10^6^/µL)	7.788 ± 0.286	7.587 ± 0.402	7.347 ± 0.379	7.570 ± 0.355
HGB (g/dL)	14.62 ± 0.57	14.13 ± 0.82	14.11 ± 0.60	14.37 ± 0.41
HCT (%)	43.08 ± 1.94	41.82 ± 2.29	41.94 ± 1.62	42.43 ± 1.34
MCV (fL)	55.31 ± 1.00	55.11 ± 1.19	57.12 ± 2.25	56.12 ± 2.04
MCH (pg)	18.76 ± 0.28	18.65 ± 0.45	19.21 ± 0.56	19.03 ± 0.84
MCHC (g/dL)	33.93 ± 0.37	33.80 ± 0.33	33.67 ± 0.73	33.90 ± 0.56
RDW (%)	11.26 ± 0.26	11.30 ± 0.42	11.65 ± 0.74	11.45 ± 0.31
HDW (g/dL)	2.187 ± 0.125	2.219 ± 0.131	2.276 ± 0.198	2.187 ± 0.105
RET (%)	2.034 ± 0.451	2.040 ± 0.324	2.429 ± 1.195	2.060 ± 0.501
PLT (10^3^/µL)	1096.8 ± 110.9	1033.2 ± 184.2	1111.1 ± 109.4	1077.0 ± 52.4
MPV (fL)	8.35 ± 0.36	8.46 ± 0.55	8.46 ± 0.19	7.99 ± 0.80
WBC (10^3^/µL)	7.184 ± 1.303	7.939 ± 2.870	8.454 ± 2.965	7.544 ± 2.327
NEU (%)	10.37 ± 2.89	14.32 ± 8.29	11.20 ± 4.07	10.03 ± 4.63
NEU (10^3^/µL)	0.742 ± 0.224	1.278 ± 1.321	0.868 ± 0.253	0.692 ± 0.241
LYM (%)	85.68 ± 3.74	81.68 ± 8.45	84.80 ± 4.49	85.01 ± 4.37
LYM (10^3^/µL)	6.164 ± 1.223	6.340 ± 1.870	7.250 ± 2.770	6.474 ± 2.250
MONO (%)	2.00 ± 0.57	2.30 ± 0.77	2.26 ± 1.12	2.94 ± 0.47
MONO (10^3^/µL)	0.141 ± 0.036	0.187 ± 0.102	0.190 ± 0.107	0.222 ± 0.066
EOS (%)	1.30 ± 0.62	1.10 ± 0.49	0.97 ± 0.35	1.22 ± 0.35
EOS (10^3^/µL)	0.092 ± 0.045	0.088 ± 0.057	0.077 ± 0.030	0.091 ± 0.034
BASO (%)	0.14 ± 0.05	0.14 ± 0.05	0.16 ± 0.08	0.14 ± 0.07
BASO (10^3^/µL)	0.010 ± 0.005	0.010 ± 0.007	0.015 ± 0.011	0.011 ± 0.010
LUC (%)	0.54 ± 0.27	0.42 ± 0.15	0.60 ± 0.18	0.67 ± 0.39
LUC (10^3^/µL)	0.037 ± 0.016	0.035 ± 0.018	0.051 ± 0.029	0.055 ± 0.055
APTT (sec)	13.33 ± 0.93	14.13 ± 1.49	14.34 ± 1.10	13.42 ± 1.06
PT (sec)	7.63 ± 0.21	7.69 ± 0.30	7.58 ± 0.18	7.58 ± 0.14
No. of animals	10	10	10	10

RBC, red blood cell; HGB, hemoglobin; HCT, hematocrit; MCV; mean corpuscular volume; MCH, mean corpuscular hemoglobin; MCHC, mean corpuscular hemoglobin concentration; RDW, red cell distribution width; HDW, hemoglobin distribution width; RET, reticulocyte; PLT, platelet; MPV, mean platelet volume; WBC, white blood cell; NEU, neutrophils; LYM, lymphocytes; MONO, monocytes; EOS, eosinophils; BASO, basophils; LUC, large unstained cell, APTT, activated partial thromboplastin time; PT, prothrombin time. Significant differences were compared with the control group.

**Table 9:** Serum biochemical values in 13 weeks repeated oral dose toxicity of WG extract in male rats.

Parameters	Dose (mg/kg/day)			
	Control	1250	2500	5000
Male				
AST (IU/L)	94.30 ± 15.17	96.50 ± 22.04	98.04 ± 17.28	102.86 ± 30.99
ALT (IU/L)	26.52 ± 5.47	28.05 ± 3.97	27.79 ± 2.61	29.61 ± 7.25
ALP (IU/L)	81.12 ± 17.57	85.80 ± 15.09	75.75 ± 13.70	80.65 ± 16.34
CPK (IU/L)	373.2 ± 186.4	352.0 ± 203.6	319.8 ± 166.0	369.7 ± 258.9
TBIL (mg/dL)	0.1569 ± 0.0299	0.1394 ± 0.0206	0.1540 ± 0.0308	0.1605 ± 0.0267
GLU (mg/dL)	148.26 ± 26.75	135.70 ± 18.55	136.94 ± 18.61	142.41 ± 18.87
TCHO (mg/dL)	76.0 ± 20.4	73.8 ± 20.6	72.5 ± 25.2	70.1 ± 19.6
TG (mg/dL)	93.2 ± 62.2	78.7 ± 25.9	82.2 ± 35.5	85.2 ± 21.8
TP (g/dL)	6.289 ± 0.305	6.189 ± 0.270	6.005 ± 0.319	6.300 ± 0.282
ALB (g/dL)	2.980 ± 0.149	2.946 ± 0.104	2.848 ± 0.169	2.956 ± 0.107
A/G Ratio	0.901 ± 0.039	0.911 ± 0.028	0.903 ± 0.028	0.886 ± 0.033
BUN (mg/dL)	12.14 ± 0.78	11.02 ± 1.30	11.47 ± 2.10	10.28 ± 1.52
CREA (mg/dL)	0.425 ± 0.042	0.418 ± 0.023	0.428 ± 0.034	0.416 ± 0.035
IP (mg/dL)	5.905 ± 0.491	5.812 ± 0.386	5.729 ± 0.385	5.831 ± 0.326
Ca (mg/dL)	10.171 ± 0.279	10.178 ± 0.222	10.015 ± 0.285	10.139 ± 0.322
Na (mmol/L)	138.526 ± 0.916	139.004 ± 0.994	140.314 ± 1.399 **	139.969 ± 0.596 **
K (mmol/L)	4.523 ± 0.341	4.546 ± 0.178	4.395 ± 0.278	4.432 ± 0.234
Cl (mmol/L)	100.825 ± 1.505	101.321 ± 1.064	101.794 ± 1.537	100.686 ± 1.791
No. of animals	10	10	10	10

AST, aspartate aminotransferase; ALT, alanine aminotransferase; ALP, alkaline phosphatase; CPK, creatine phosphokinase; TBIL, total bilirubin; GLU, glucose; TCHO, total cholesterol; TG, triglyceride; TP, total protein; ALB, albumin; A/G, albumin/globulin; BUN, blood urea nitrogen; CREA, creatinine; IP, inorganic phosphorus; Ca, calcium; Na, sodium; K, potassium ion; Cl, chloride ion. Significant differences were compared with the control group, ** *P* < 0.01.

**Table 10:** Serum biochemical values in 13 weeks repeated oral dose toxicity of WG extract in female rats.

Parameters	Dose (mg/kg/day)			
	Control	1250	2500	5000
Female				
AST (IU/L)	92.39 ± 40.42	121.73 ± 98.49	97.90 ± 38.84	92.33 ± 23.21
ALT (IU/L)	26.07 ± 6.72	36.11 ± 39.39	27.88 ± 11.12	24.07 ± 4.37
ALP (IU/L)	33.90 ± 8.57	37.48 ± 9.41	38.96 ± 14.05	47.66 ± 10.38 *
CPK (IU/L)	234.1 ± 170.5	237.0 ± 125.5	247.6 ± 134.9	251.3 ± 153.6
TBIL (mg/dL)	0.2024 ± 0.0181	0.2083 ± 0.0272	0.2166 ± 0.0577	0.1884 ± 0.0252
GLU (mg/dL)	120.23 ± 13.89	120.25 ± 7.89	130.79 ± 15.62	126.40 ± 18.62
TCHO (mg/dL)	80.1 ± 15.5	88.9 ± 31.6	78.1 ± 20.4	72.3 ± 16.8
TG (mg/dL)	31.4 ± 7.6	35.2 ± 7.1	37.3 ± 8.2	37.0 ± 13.1
TP (g/dL)	6.643 ± 0.404	6.736 ± 0.498	6.813 ± 0.376	6.584 ± 0.303
ALB (g/dL)	3.518 ± 0.260	3.498 ± 0.317	3.536 ± 0.264	3.402 ± 0.211
A/G Ratio	1.127 ± 0.055	1.079 ± 0.048	1.079 ± 0.066	1.069 ± 0.056
BUN (mg/dL)	15.16 ± 2.75	13.52 ± 2.61	14.15 ± 2.71	14.85 ± 1.92
CREA (mg/dL)	0.497 ± 0.054	0.509 ± 0.041	0.552 ± 0.081	0.538 ± 0.057
IP (mg/dL)	5.545 ± 0.440	5.616 ± 0.446	5.355 ± 0.332	5.427 ± 0.290
Ca (mg/dL)	10.422 ± 0.340	10.479 ± 0.331	10.302 ± 0.428	10.236 ± 0.297
Na (mmol/L)	136.228 ± 1.378	136.250 ± 1.150	136.242 ± 0.942	137.330 ± 0.949
K (mmol/L)	3.850 ± 0.341	3.995 ± 0.183	3.779 ± 0.222	3.763 ± 0.219
Cl (mmol/L)	100.936 ± 1.173	100.298 ± 0.764	100.117 ± 1.849	100.866 ± 1.598
No. of animals	10	10	10	10

AST, aspartate aminotransferase; ALT, alanine aminotransferase; ALP, alkaline phosphatase; CPK, creatine phosphokinase; TBIL, total bilirubin; GLU, glucose; TCHO, total cholesterol; TG, triglyceride; TP, total protein; ALB, albumin; A/G, albumin/globulin; BUN, blood urea nitrogen; CREA, creatinine; IP, inorganic phosphorus; Ca, calcium; Na, sodium; K, potassium ion; Cl, chloride ion. Significant differences were compared with the control group, * *P* < 0.05.

**Table 11:** Absolute and relative organ weights in 13 weeks repeated oral dose toxicity of WG extract in male rats.

Parameters	Dose (mg/kg/day)			
	Control	1250	2500	5000
Male				
Bodyweights (g) ^(a)^	607.806 ± 46.392	594.079 ± 41.628	598.491 ± 58.535	591.249 ± 55.237
Brain (g)	2.230 ± 0.100	2.194 ± 0.073	2.219 ± 0.093	2.227 ± 0.117
% to bodyweight	0.368 ± 0.023	0.370 ± 0.020	0.374 ± 0.036	0.378 ± 0.022
Pituitary Gland	0.015 ± 0.003	0.014 ± 0.003	0.015 ± 0.003	0.014 ± 0.002
% to bodyweight	0.002 ± 0.000	0.002 ± 0.000	0.003 ± 0.001	0.002 ± 0.000
Lung	1.888 ± 0.130	1.903 ± 0.175	1.902 ± 0.088	1.953 ± 0.275
% to bodyweight	0.311 ± 0.019	0.321 ± 0.028	0.321 ± 0.037	0.330 ± 0.029
Heart	1.704 ± 0.102	1.661 ± 0.175	1.664 ± 0.101	1.757 ± 0.213
% to bodyweight	0.281 ± 0.016	0.280 ± 0.023	0.280 ± 0.024	0.297 ± 0.025
Thymus	0.400 ± 0.095	0.379 ± 0.078	0.315 ± 0.066	0.381 ± 0.102
% to bodyweight	0.066 ± 0.015	0.064 ± 0.012	0.053 ± 0.009	0.064 ± 0.013
Spleen	1.018 ± 0.165	0.929 ± 0.207	1.039 ± 0.196	0.965 ± 0.181
% to bodyweight	0.168 ± 0.029	0.155 ± 0.026	0.175 ± 0.034	0.163 ± 0.021
Adrenal (left)	0.033 ± 0.006	0.032 ± 0.007	0.032 ± 0.003	0.030 ± 0.006
% to bodyweight	0.005 ± 0.001	0.005 ± 0.001	0.005 ± 0.001	0.005 ± 0.001
Adrenal (right)	0.030 ± 0.007	0.031 ± 0.005	0.030 ± 0.003	0.029 ± 0.007
% to bodyweight	0.005 ± 0.001	0.005 ± 0.001	0.005 ± 0.001	0.005 ± 0.001
Kidney (left)	1.707 ± 0.187	1.699 ± 0.169	1.732 ± 0.183	1.869 ± 0.232
% to bodyweight	0.282 ± 0.031	0.286 ± 0.020	0.291 ± 0.028	0.316 ± 0.032 *
Kidney (right)	1.722 ± 0.188	1.703 ± 0.137	1.705 ± 0.206	1.839 ± 0.186
% to bodyweight	0.284 ± 0.032	0.287 ± 0.017	0.286 ± 0.030	0.311 ± 0.024
Liver	15.778 ± 2.144	14.926 ± 1.693	14.962 ± 2.000	15.924 ± 1.892
% to bodyweight	2.595 ± 0.291	2.507 ± 0.146	2.499 ± 0.198	2.693 ± 0.189
Testis (left)	1.894 ± 0.107	2.046 ± 0.216	1.880 ± 0.065	1.984 ± 0.182
% to bodyweight	0.313 ± 0.031	0.345 ± 0.034	0.317 ± 0.033	0.338 ± 0.042
Testis (right)	1.885 ± 0.109	2.053 ± 0.198 *	1.884 ± 0.096	1.976 ± 0.176
% to bodyweight	0.312 ± 0.031	0.346 ± 0.033	0.318 ± 0.038	0.336 ± 0.041
Epididymis (left)	0.773 ± 0.067	0.760 ± 0.079	0.781 ± 0.069	0.784 ± 0.049
% to bodyweight	0.128 ± 0.011	0.128 ± 0.010	0.131 ± 0.008	0.133 ± 0.011
Epididymis (right)	0.772 ± 0.051	0.782 ± 0.061	0.789 ± 0.070	0.825 ± 0.071
% to bodyweight	0.128 ± 0.012	0.132 ± 0.011	0.132 ± 0.010	0.140 ± 0.009
Prostate Gland	0.622 ± 0.134	0.680 ± 0.200	0.725 ± 0.188	0.760 ± 0.181
% to bodyweight	0.102 ± 0.019	0.115 ± 0.035	0.121 ± 0.028	0.129 ± 0.031
No. of animals	10	10	10	10

^(a)^ Bodyweights were measured immediately prior to necropsy after an overnight fast. Significant differences were compared with the control group.

**Table 12:** Absolute and relative organ weights in 13 weeks repeated oral dose toxicity of WG extract in female rats.

Parameters	Dose (mg/kg/day)			
	Control	1250	2500	5000
Female				
Bodyweights (g) ^(a)^	257.329 ± 12.846	273.903 ± 24.745	292.277 ± 37.093 *	271.045 ± 20.724
Brain	1.957 ± 0.093	2.030 ± 0.078	2.017 ± 0.048	2.023 ± 0.093
% to bodyweight	0.763 ± 0.062	0.746 ± 0.072	0.699 ± 0.080	0.751 ± 0.076
Pituitary	0.018 ± 0.003	0.019 ± 0.003	0.021 ± 0.005	0.019 ± 0.003
% to bodyweight	0.007 ± 0.001	0.007 ± 0.001	0.007 ± 0.002	0.007 ± 0.001
Lung	1.121 ± 0.154	1.245 ± 0.067 *	1.274 ± 0.114 *	1.225 ± 0.086
% to bodyweight	0.436 ± 0.059	0.458 ± 0.046	0.439 ± 0.046	0.454 ± 0.046
Heart	0.981 ± 0.157	0.958 ± 0.088	0.982 ± 0.133	0.933 ± 0.088
% to bodyweight	0.382 ± 0.066	0.351 ± 0.029	0.336 ± 0.023	0.344 ± 0.021
Thymus	0.267 ± 0.044	0.262 ± 0.064	0.274 ± 0.072	0.271 ± 0.053
% to bodyweight	0.104 ± 0.021	0.095 ± 0.020	0.094 ± 0.021	0.100 ± 0.016
Spleen	0.454 ± 0.046	0.481 ± 0.065	0.527 ± 0.152	0.493 ± 0.074
% to bodyweight	0.177 ± 0.019	0.176 ± 0.025	0.180 ± 0.043	0.183 ± 0.032
Adrenal (left)	0.032 ± 0.005	0.035 ± 0.007	0.035 ± 0.006	0.036 ± 0.007
% to bodyweight	0.012 ± 0.002	0.013 ± 0.002	0.012 ± 0.002	0.013 ± 0.003
Adrenal (right)	0.031 ± 0.004	0.033 ± 0.006	0.035 ± 0.007	0.034 ± 0.005
% to bodyweight	0.012 ± 0.002	0.012 ± 0.002	0.012 ± 0.002	0.013 ± 0.002
Kidney (left)	0.830 ± 0.076	0.877 ± 0.078	0.920 ± 0.150	0.890 ± 0.092
% to bodyweight	0.324 ± 0.038	0.322 ± 0.037	0.315 ± 0.028	0.329 ± 0.032
Kidney (right)	0.854 ± 0.067	0.885 ± 0.072	0.938 ± 0.151	0.915 ± 0.095
% to bodyweight	0.333 ± 0.032	0.325 ± 0.033	0.321 ± 0.028	0.338 ± 0.029
Liver	6.476 ± 0.617	7.097 ± 1.090	7.528 ± 1.188	7.196 ± 0.918
% to bodyweight	2.516 ± 0.189	2.586 ± 0.275	2.572 ± 0.168	2.651 ± 0.202
Ovary (left)	0.039 ± 0.015	0.036 ± 0.012	0.036 ± 0.009	0.036 ± 0.005
% to bodyweight	0.015 ± 0.006	0.013 ± 0.004	0.012 ± 0.003	0.013 ± 0.003
Ovary (right)	0.038 ± 0.016	0.038 ± 0.014	0.033 ± 0.010	0.07 ± 0.010
% to bodyweight	0.015 ± 0.006	0.014 ± 0.005	0.011 ± 0.004	0.014 ± 0.004
Uterus and Cervix	0.649 ± 0.222	0.700 ± 0.293	0.722 ± 0.180	0.569 ± 0.157
% to bodyweight	0.253 ± 0.089	0.257 ± 0.115	0.249 ± 0.065	0.208 ± 0.044
No. of animals	10	10	10	10

^(a)^ Bodyweights were measured immediately prior to necropsy after an overnight fast. Significant differences were compared with the control group, * *P* < 0.05.

**Table 13:** Necropsy findings in 13 weeks repeated oral dose toxicity of WG extract in male rats.

Parameters	Observation Type	Dose (mg/kg/day)			
		Control	1250	2500	5000
Male					
Adrenal Gland	Normal	10	10	10	10
Aorta	Normal	10	10	10	10
Bone Marrow, Sternum	Normal	10	10	10	10
Brain	Normal	10	10	10	10
Cervix	Normal	-	-	-	-
Coagulating Gland	Normal	10	10	10	10
Epididymis	Normal	10	10	10	10
Esophagus	Normal	10	10	10	10
Eye with Optic Nerve	Normal	10	10	10	10
Harderian Gland	Normal	10	10	10	10
Heart	Normal	10	10	10	10
Cecum	Normal	10	10	10	10
Colon	Normal	10	10	10	10
Duodenum	Normal	10	10	10	10
Ileum	Normal	10	10	10	10
Jejunum	Normal	10	10	10	10
Rectum	Normal	10	10	10	10
Kidney	Normal	10	10	10	10
Liver	Normal	10	10	10	10
	Discoloration	0	0	0	0
Lung	Normal	10	10	10	10
Lymph Node, Mesenteric	Normal	10	10	10	10
Lymph Node, Mandibular	Normal	10	10	10	10
Skeletal Muscle	Normal	10	10	10	10
Nerve, Peripheral	Normal	10	10	10	10
Ovary	Normal	-	-	-	-
Pancreas	Normal	10	10	10	10
Parathyroid Gland	Normal	9	10	10	10
	Absent	1	0	0	0
Pituitary Gland	Normal	10	10	10	10
Prostate Gland	Normal	10	10	10	10
Salivary Gland	Normal	10	10	10	10
Seminal Vesicle	Normal	10	10	10	10
Skin	Normal	10	10	10	9
	Alopecia	0	0	0	1
	Crust	0	0	0	1
Skin, Mammary	Normal	10	10	10	10
Spinal Cord, Thoracic	Normal	10	10	10	10
Spleen	Normal	10	10	10	10
Stomach	Normal	10	10	10	10
Testis	Normal	10	10	10	10
Thymus	Normal	10	10	10	10
Thyroid Gland	Normal	9	10	10	10
	Enlarged	1	0	0	0
	Absent	1	0	0	0
Tongue	Normal	10	10	10	10
Trachea	Normal	10	10	10	10
Urinary Bladder	Normal	10	10	10	10
Uterus	Normal	-	-	-	-
Vagina	Normal	-	-	-	-
Femorotibial Joint	Normal	10	10	10	10
No. of animals		10	10	10	10

-: Not applicable.

**Table 14:** Necropsy findings in 13 weeks repeated oral dose toxicity of WG extract in female rats.

Parameters	Observation Type	Dose (mg/kg/day)			
		Control	1250	2500	5000
Female					
Adrenal Gland	Normal	10	10	10	10
Aorta	Normal	10	10	10	10
Bone Marrow, Sternum	Normal	10	10	10	10
Brain	Normal	10	10	10	10
Coagulating Gland	Normal	-	-	-	-
Epididymis	Normal	-	-	-	-
Esophagus	Normal	10	10	10	10
Eye with Optic Nerve	Normal	10	10	10	10
Harderian Gland	Normal	10	10	10	10
Heart	Normal	10	10	10	10
Cecum	Normal	10	10	10	10
Colon	Normal	10	10	10	10
Duodenum	Normal	10	10	10	10
Ileum	Normal	10	10	10	10
Jejunum	Normal	10	10	10	10
Rectum	Normal	10	10	10	10
Kidney	Normal	10	10	10	10
Liver	Normal	10	9	10	10
	Discoloration	10	1	10	10
Lung	Normal	10	10	10	10
Lymph Node, Mesenteric	Normal	10	10	10	10
Lymph Node, Mandibular	Normal	10	10	10	10
Skeletal Muscle	Normal	10	10	10	10
Nerve, Peripheral	Normal	10	10	10	10
Ovary	Normal	10	10	10	10
Pancreas	Normal	10	10	10	10
Parathyroid Gland	Normal	10	10	10	10
	Absent	0	0	0	0
Pituitary Gland	Normal	10	10	10	10
Prostate Gland	Normal	-	-	-	-
Salivary Gland	Normal	10	10	10	10
Seminal Vesicle	Normal	-	-	-	-
Skin	Normal	10	10	10	10
	Alopecia	0	0	0	0
	Crust	0	0	0	0
Skin, Mammary	Normal	10	10	10	10
Spinal Cord, Thoracic	Normal	10	10	10	10
Spleen	Normal	10	10	10	10
Stomach	Normal	10	10	10	10
Testis	Normal	-	-	-	-
Thymus	Normal	10	10	10	10
Thyroid Gland	Normal	10	10	10	10
	Enlarged	0	0	0	0
	Absent	0	0	0	0
Tongue	Normal	10	10	10	10
Trachea	Normal	10	10	10	10
Urinary Bladder	Normal	10	10	10	10
Vagina	Normal	10	10	10	10
Femorotibial Joint	Normal	10	10	10	10
Uterus and Cervix	Normal	9	7	8	9
	Retention	1	3	2	1
No. of animals		10	10	10	10

-: Not applicable.

## Data Availability

Datasets used and/or analyzed in the current study are available from the corresponding author on reasonable request.

## Supplementary Materials

The following are available online at https://www.mdpi.com/article/10.3390/plants10061126/s1, Figure S1: Bodyweight of male and female rats in the 4 weeks repeated oral dose toxicity study of WG extract, Figure S2: Food consumption of male and female rats in the 4 weeks repeated oral dose toxicity study of WG extract, Figure S3: Water consumption of male and female rats in the 4 weeks repeated oral dose toxicity study of WG extract, Table S1: Mortality, Clinical signs and Ophthalmological examination in 4 weeks repeated oral dose toxicity of WG, Table S2: Urinalysis in 4 weeks repeated oral dose toxicity of WG in male rats, Table S3: Urinalysis in 4 weeks repeated oral dose toxicity of WG in female rats, Table S4: Hematology values in 4 weeks repeated oral dose toxicity of WG in male rats, Table S5: Hematology values in 4 weeks repeated oral dose toxicity of WG in female rats, Table S6: Serum biochemical values in 4 weeks repeated oral dose toxicity of WG in male rats, Table S7: Serum biochemical values in 4 weeks repeated oral dose toxicity of WG in female rats, Table S8: Absolute and relative organ weights in 4 weeks repeated oral dose toxicity of WG in male rats, Table S9: Absolute and relative organ weights in 4 weeks repeated oral dose toxicity of WG in female rats, Table S10: Necropsy findings in 4 weeks repeated oral dose toxicity of WG in male rats, Table S11: Necropsy findings in 4 weeks repeated oral dose toxicity of WG in female rats.
