# Supplementary material for: Assessment of General Toxicity of the Glycyrrhiza New Variety Extract in Rats
Source: Plants (Basel). 2021 Jun 1;10(6):1126. doi: 10.3390/plants10061126 (PMC8229005; doi:10.3390/plants10061126)
Supplement: Supplementary file 1 [file plants-10-01126-s001.zip › plants-1231393-Final Supplementary Materials and Methods (Plants).pdf]

Supplementary Materials and Methods

1. Wongam Preparation

WG was obtained from the Korean Rural Development Administration, and extracted by Wonkwang Herb Co. (Jinan-gun, Jeollabuk-do, Republic of Korea). Briefly, WG was extracted with distilled water at 100℃ for 4 h 30 min. The extract was concentrated under reduced pressure in a rotary evaporator at 70℃ for 3 h. The decoction was filtered using Whatman filter paper no.1. Then, it was lyophilized (Batch methods) and stored at 4℃. The yield of the dried extract from the starting crude of the WG was 8.8 %. The WG extract was prepared for administration by suspending with sterile water for injection according to the doses assigned for each group in the single and repeated oral dose toxicity studies.

2. Experimental animals and animal husbandry

All toxicological studies were carried out by Chemon Inc. under Good Laboratory Practice (GLP) conditions. Specific pathogen-free Sprague-Dawley rats were obtained from Orient bio Inc. (Gapyeong, Gyeonggi-do, Republic of Korea). The studies were approved by the Institutional Animal Care and Use Committee (IACUC) of the Preclinical Research Center, Chemon Inc. (Approval Number: 19-R512). Animals were housed in the laboratory animal facility at a temperature of 23 ± 3°C and a relative humidity of 55 ± 15%. Animal housing was maintained under a 12-hour light-dark cycle, with 10-20 air changes per hour. Animals were supplied irradiation-sterilized pellet feed (Teklad Certified Irradiated Global 18% Protein Rodent Diet, 2918C; Envigo RMS, Inc., IN, USA), along with tap water disinfected using an ultraviolet sterilizer and ultrafiltration *ad libitum*. All animals were acclimated for 6 days before the start of the experiment. This study was conducted in accordance with test guidelines from the Korean Ministry of Food and Drug Safety (MFDS, 2018) and guidelines for the testing of chemicals from the Organization for Economic Cooperation and Development (OECD, 1997) under GLP Regulations.

3. 4-week repeated oral dose toxicity study

The high dose was set at 5000 mg/kg/day, based the absence of toxic signs in a single dose study. 6-week-old male and female Sprague-Dawley rats (n = 5 per sex and group) were orally administered with WG at 0, 625, 1250, 2500 and 5000 mg/kg/day for 4 weeks. Body weight ranges at the initiation of dosing were 193.24-213.11 g for males and 146.52-169.26 g for females, respectively.

Animals were individually checked once a day to observe any clinical signs and mortalities, and the type, date of occurrence and severity of signs were recorded. Body weights of all group rats were recorded before the initiation of dosing (day 1) and once a week during the experimental period. Before necropsy, all rat groups were fasted overnight and body weights were recorded at necropsy. Food and water intake was checked on the same days as body weight measurements were recorded. Ophthalmological observation was macroscopically evaluated.

Urinalysis was performed during the last week of observation; all animals were individually housed in a stainless-steel cage cleaned and disinfected with 70% alcohol. Urine samples were collected and 0.3 mL of fresh urine was taken for analysis. Urine samples were analyzed for glucose, bilirubin, ketone body, specific gravity, pH, protein, urobilinogen, nitrite, occult blood, leucocytes, urine color and clarity using an automatic analyzer (Clinitek Advantus; Siemens, Munchen, Germany).

For necropsy, animals were euthanized by inhalation of 3-5% isoflurane (Terel liquid; Kyongbo Pharma. Co., Ltd., Asan-si, Chungcheongnam-do, Republic of Korea) at Day 28. Blood samples were collected from the posterior vena cava for hematological and serum biochemical testing. Approximately 1 mL blood was placed in a CBC bottle (Vacutainer 3 mL; BD, NJ, USA) with anticoagulant EDTA-2K. Hematology parameters were measured using a hematology analyzer (ADVIA 2120; Siemens, Munchen, Germany), including RBC, hemoglobin (HGB), hematocrit (HCT), mean corpuscular volume (MCV), mean corpuscular hemoglobin (MCH), mean corpuscular hemoglobin concentration (MCHC), red cell distribution width (RDW), hemoglobin distribution width (HDW), platelet count (PLT), mean platelet volume (MPV), white blood cell count (WBC), neutrophils count (NEU), lymphocytes count (LYM), monocytes count (MONO), eosinophils count (EOS), basophils count (BASO), and large unstained cell count (LUC).

Serum biochemical parameters were measured using a serum biochemistry analyzer (AU680; Beckman Coulter, CA, USA). About 2 mL of the blood sample was added to a 5 mL Vacutainer tube (SST™ II Advance; BD, NJ, USA) containing clot activator. The blood was coagulated by being left at room temperature for 15-20 minutes and then centrifuged for 10 minutes (3000 rpm, 1902 Relative Centrifugal Force (RCF), Combi-514R; Hanil, Daejeon, Republic of Korea) to collect serum sample. Parameters examined were aspartate aminotransferase (AST), alanine aminotransferase (ALT), alkaline phosphatase (ALP), creatine phosphokinase (CPK), total bilirubin (TBIL), glucose (GLU), total cholesterol (TCHO), triglyceride (TG), total protein (TP), albumin (ALB), albumin/globulin (A/G) ratio, blood urea nitrogen (BUN), creatinine (CREA), inorganic phosphorus (IP), calcium (Ca), sodium (Na), potassium ion (K) and chloride ion (Cl).

After blood sampling, animals were sacrificed by exsanguination from the abdominal aorta and posterior vena cava. Gross findings were recorded, including body surface, subcutis, head and all organs in the abdominal and thoracic cavities. Following this, organs were weighed using an electronic balance (Sartorius AG): kidney (both), spleen, liver, thymus, heart, lung and brain. Organ weights (%) relative to terminal body weights were also calculated.

4. Statistical Analysis

Data are presented as mean ± standard deviation. Statistical analysis was performed by parametric one-way analysis of variance (ANOVA), and significant differences between the vehicle control and treatment groups was estimated by Dunnett's test using the ProvantisTM 10.10.1 package. The level of significance was taken as $P < 0.05$.

In this study, unless specified otherwise, the term "significant" in the sentence with P-value implies that inter-group differences have attained statistical significance compared to the control group.

Supplementary Materials and Methods

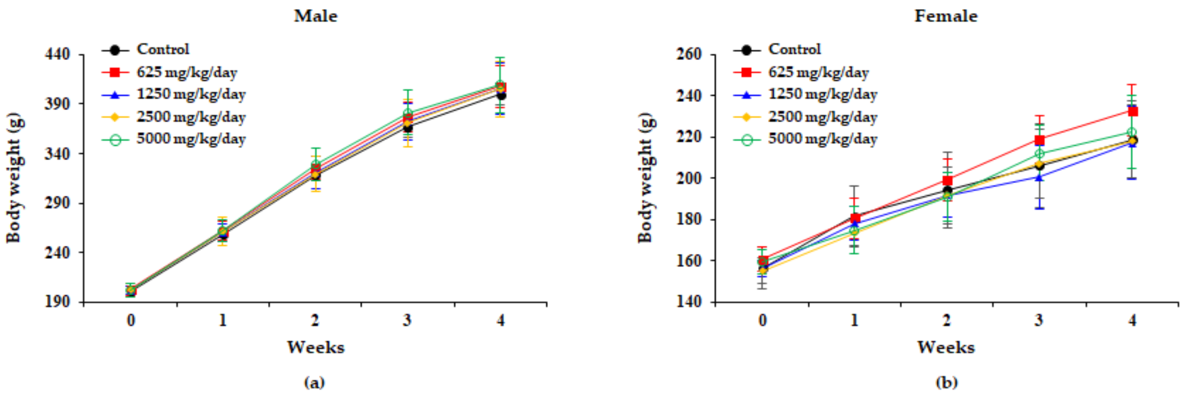

Figure S1. Body weight of male (a) and female (b) rats treated with 0, 625, 1250, 2500 and 5000 mg/kg/day in the 4 weeks repeated oral dose toxicity study of WG extract. Results are presented as the mean ± standard deviation (n = 5). Significantly different from the control group at $*P < 0.05$, $**P < 0.01$, $***P < 0.001$.

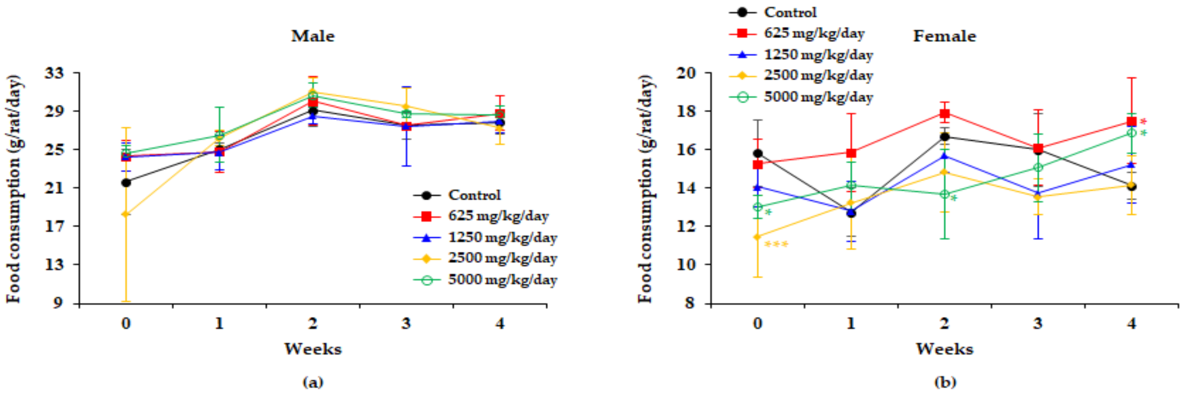

Figure S2. Food consumption of male (a) and female (b) rats treated with 0, 625, 1250, 2500 and 5000 mg/kg/day in the 4 weeks repeated oral dose toxicity study of WG extract. Results are presented as the mean ± standard deviation (n = 5). Significantly different from the control group at $*P < 0.05$, $**P < 0.01$, $***P < 0.001$.

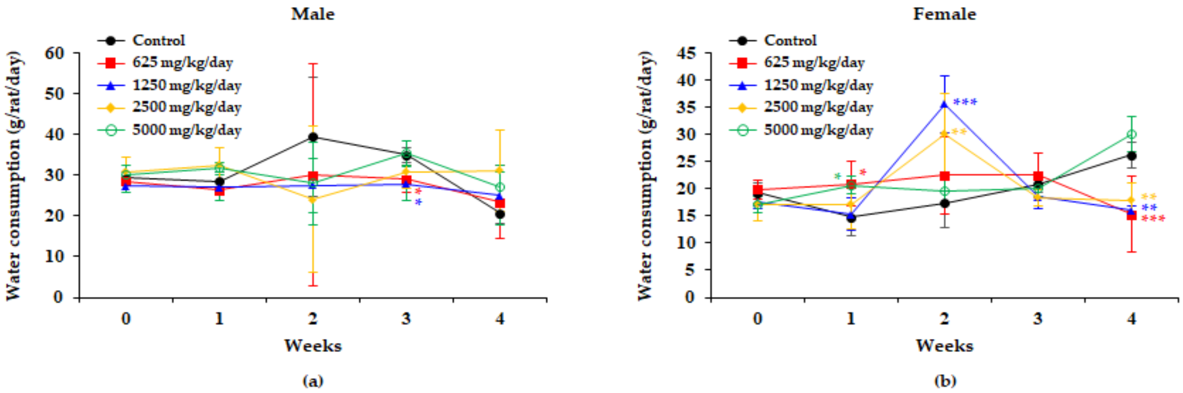

Figure S3. Water consumption of male (a) and female (b) rats treated with 0, 625, 1250, 2500 and 5000 mg/kg/day in the 4 weeks repeated oral dose toxicity study of WG extract. Results are presented as the mean ± standard deviation (n = 5). Significantly different from the control group at $*P < 0.05$, $**P < 0.01$, $***P < 0.001$.

Table S1. Mortality, Clinical signs and Ophthalmological examination in 4 weeks repeated oral dose toxicity of WG extract

	From Day 1 to Day 28

Observation Type	Male					Female				
	Dose (mg/kg/day)					Dose (mg/kg/day)				
	Control	625	1250	2500	5000	Control	625	1250	2500	5000
Normal	5	5	0	0	0	5	5	0	0	0
Salivation	0	0	0	0	3	0	0	0	0	0
Loss of fur	0	0	1	0	0	0	0	0	0	0
Compound-colored stool	0	0	5	5	5	0	0	5	5	5
Ophthalmological examination	N	N	N	N	N	N	N	N	N	N
Mortality (dead/total)	0% (0/5)	0% (0/5)	0% (0/5)	0% (0/5)	0% (0/5)	0% (0/5)	0% (0/5)	0% (0/5)	0% (0/5)	0% (0/5)
No. of animals	5	5	5	5	5	5	5	5	5	5

N, Normal.

Table S2. Urinalysis in 4 weeks repeated oral dose toxicity of WG extract in male rats

Parameters	Result	Dose (mg/kg/day)				
		Control	625	1250	2500	5000
Male						
Protein	Negative	5	3	2	4	1
	trace	0	2	2	1	2
	1+	0	0	1	0	2
Ketones	Negative	4	3	4	3	1
	trace	1	2	1	2	2
	1+	0	0	0	0	2
Glucose	Negative	5	5	5	5	5
Bilirubin	Negative	5	5	5	5	5
Blood	Negative	4	5	4	5	5
	Trace	0	0	1	0	0
	2+	1	0	0	0	0
Urobilinogen	0.2 EU/dL	5	5	5	5	5
pH	7.0	0	0	0	0	0
	7.5	0	0	0	0	0
	8.0	1	0	0	1	1
	8.5	4	5	5	4	4
Nitrites	Negative	5	5	5	5	5
	Positive	0	0	0	0	0
Specific Gravity	≤1.005	0	0	0	0	0
	1.010	4	3	5	4	2
	1.015	1	2	0	0	2
	1.020	0	0	0	1	1

Leucocytes	Negative	5	5	5	5	3
	Trace	0	0	0	0	2
Colour	Yellow	5	5	5	5	5
Clarity	Clear	5	5	5	5	5
No. of animals		5	5	5	5	5

RBC, red blood cell; WBC, white blood cell.
[a] Values are mean ± standard deviation. Significant differences were compared with the control group, $^{*}P < 0.05$, $^{**}P < 0.01$, $^{***}P < 0.001$.

Table S3. Urinalysis in 4 weeks repeated oral dose toxicity of WG extract in female rats

Parameters	Result	Dose (mg/kg/day)				
		Control	625	1250	2500	5000
Female						
Protein	Negative	5	5	5	5	4
	trace	0	0	0	0	1
	1+	0	0	0	0	0
Ketones	Negative	5	5	5	5	0
	trace	0	0	0	0	3
	1+	0	0	0	0	2
Glucose	Negative	5	5	5	5	5
Bilirubin	Negative	5	5	5	5	5
Blood	Negative	4	5	5	5	5
	Trace	0	0	0	0	0
	2+	1	0	0	0	0
Urobilinogen	0.2	5	5	5	5	5
pH	7.0	1	0	0	0	0
	7.5	0	1	0	0	0
	8.0	0	1	1	2	1
	8.5	4	3	4	3	4
Nitrites	Negative	5	5	5	4	5
	Positive	0	0	0	1	0
Specific Gravity	≤1.005	1	1	1	1	0
	1.010	4	4	4	4	3
	1.015	0	0	0	0	1
	1.020	0	0	0	0	1
Leucocytes	Negative	5	5	5	5	4
	Trace	0	0	0	0	1
Colour	Yellow	5	5	5	5	5
Clarity	Clear	5	5	5	5	5
No. of animals		5	5	5	5	5

RBC, red blood cell; WBC, white blood cell.

a) Values are mean ± standard deviation. Significant differences were compared with the control group, $*P < 0.05$, $**P < 0.01$, $***P < 0.001$.

Table S4. Hematology values in 4 weeks repeated oral dose toxicity of WG extract in male rats

Parameters	Dose (mg/kg/day)				
	Control	625	1250	2500	5000
Male					
RBC (10^6/μL)	7.786 ± 0.530	7.716 ± 0.144	7.784 ± 0.205	7.532 ± 0.222	7.700 ± 0.361
HGB (g/dL)	15.00 ± 0.59	14.90 ± 0.39	14.68 ± 0.35	14.78 ± 0.32	14.92 ± 0.75
HCT (%)	47.10 ± 1.79	46.82 ± 1.70	46.46 ± 1.30	46.52 ± 0.96	46.70 ± 1.90
MCV (fL)	60.58 ± 2.17	60.68 ± 1.51	59.72 ± 1.91	61.84 ± 1.51	60.66 ± 1.34
MCH (pg)	19.30 ± 0.58	19.36 ± 0.25	18.88 ± 0.46	19.66 ± 0.52	19.36 ± 0.67
MCHC (g/dL)	31.84 ± 0.30	31.86 ± 0.43	31.58 ± 0.29	31.80 ± 0.29	31.94 ± 0.48
RDW (%)	11.64 ± 0.27	11.78 ± 0.31	11.42 ± 0.36	11.72 ± 0.36	11.70 ± 0.25
HDW (g/dL)	2.424 ± 0.119	2.430 ± 0.194	2.310 ± 0.103	2.512 ± 0.188	2.386 ± 0.133
PLT (10^3/μL)	1023.6 ± 73.1	1026.8 ± 88.0	1050.4 ± 153.3	1086.6 ± 125.5	994.2 ± 159.2
MPV (fL)	5.68 ± 0.18	5.52 ± 0.11	5.62 ± 0.23	5.50 ± 0.22	5.56 ± 0.25
WBC (10^3/μL)	10.878 ± 3.123	12.884 ± 1.469	10.908 ± 2.148	11.592 ± 1.049	11.024 ± 3.155
NEU (%)	14.26 ± 4.93	12.14 ± 3.17	12.14 ± 2.12	14.52 ± 2.67	14.58 ± 4.99
NEU (10^3/μL)	1.578 ± 0.715	1.542 ± 0.295	1.296 ± 0.185	1.668 ± 0.276	1.568 ± 0.640
LYM (%)	80.78 ± 5.37	83.14 ± 4.08	82.48 ± 2.30	80.30 ± 2.74	80.24 ± 5.98
LYM (10^3/μL)	8.766 ± 2.543	10.746 ± 1.618	9.022 ± 1.976	9.320 ± 1.034	8.912 ± 2.790
MONO (%)	3.44 ± 0.84	3.10 ± 0.98	3.56 ± 1.04	3.32 ± 0.55	3.64 ± 1.25
MONO (10^3/μL)	0.368 ± 0.119	0.400 ± 0.129	0.394 ± 0.145	0.386 ± 0.091	0.374 ± 0.077
EOS (%)	0.64 ± 0.29	0.68 ± 0.27	0.74 ± 0.19	0.78 ± 0.22	0.72 ± 0.23
EOS (10^3/μL)	0.068 ± 0.036	0.086 ± 0.029	0.080 ± 0.020	0.090 ± 0.023	0.078 ± 0.015
BASO (%)	0.24 ± 0.09	0.28 ± 0.11	0.30 ± 0.07	0.30 ± 0.07	0.28 ± 0.13
BASO (10^3/μL)	0.28 ± 0.019	0.036 ± 0.018	0.034 ± 0.011	0.032 ± 0.008	0.036 ± 0.019
LUC (%)	0.62 ± 0.23	0.66 ± 0.26	0.70 ± 0.16	0.80 ± 0.17	0.54 ± 0.13
LUC (10^3/μL)	0.070 ± 0.031	0.084 ± 0.026	0.076 ± 0.019	0.092 ± 0.016	0.054 ± 0.011
No. of animals	5	5	5	5	5

RBC, red blood cell; HGB, hemoglobin; HCT, hematocrit; MCV; mean corpuscular volume; MCH, mean corpuscular hemoglobin; MCHC, mean corpuscular hemoglobin concentration; RDW, red cell distribution width; HDW, hemoglobin distribution width; PLT, platelet; MPV, mean platelet volume; WBC, white blood cell; NEU, neutrophils; LYM, lymphocytes; MONO, monocytes; EOS, eosinophils; BASO, basophils; LUC, large unstained cell. Significant differences were compared with the control group, $*P < 0.05$, $**P < 0.01$, $***P < 0.001$.

Table S5. Hematology values in 4 weeks repeated oral dose toxicity of WG extract in female rats

Parameters	Dose (mg/kg/day)				
	Control	625	1250	2500	5000
Female					
RBC (10^6/μL)	7.584 ± 0.352	7.516 ± 0.311	7.662 ± 0.212	7.594 ± 0.339	7.612 ± 0.343
HGB (g/dL)	14.50 ± 0.80	14.76 ± 0.46	14.24 ± 0.46	14.46 ± 0.56	14.58 ± 0.44

HCT (%)	44.12 ± 2.31	44.24 ± 1.65	43.06 ± 1.15	43.90 ± 1.74	44.34 ± 1.26
MCV (fL)	58.16 ± 1.50	58.84 ± 1.30	56.24 ± 1.68	57.80 ± 1.51	58.28 ± 1.22
MCH (pg)	19.12 ± 0.52	19.66 ± 0.40	18.58 ± 0.66	19.08 ± 0.73	19.16 ± 0.57
MCHC (g/dL)	32.84 ± 0.26	33.34 ± 0.36	33.08 ± 0.40	32.98 ± 0.56	32.86 ± 0.34
RDW (%)	10.76 ± 0.43	10.82 ± 0.28	10.86 ± 0.43	10.68 ± 0.35	10.54 ± 0.30
HDW (g/dL)	2.258 ± 0.143	2.364 ± 0.100	2.342 ± 0.132	2.300 ± 0.124	2.228 ± 0.094
PLT (10^3/μL)	1103.6 ± 81.2	975.6 ± 46.1	1033.2 ± 125.8	1022.4 ± 69.0	1029.6 ± 65.9
MPV (fL)	5.58 ± 0.18	5.56 ± 0.17	5.46 ± 0.09	5.60 ± 0.00	5.52 ± 0.22
WBC (10^3/μL)	7.694 ± 1.365	9.132 ± 1.541	6.760 ± 1.855	7.534 ± 1.412	6.906 ± 0.486
NEU (%)	16.64 ± 6.09	14.36 ± 5.13	21.40 ± 5.11	12.28 ± 2.74	12.78 ± 1.93
NEU (10^3/μL)	1.230 ± 0.316	1.282 ± 0.409	1.432 ± 0.423	0.904 ± 0.117	0.874 ± 0.083
LYM (%)	79.44 ± 6.17	80.86 ± 5.93	73.56 ± 5.48	83.14 ± 3.15	82.92 ± 2.86
LYM (10^3/μL)	6.162 ± 1.475	7.420 ± 1.527	4.990 ± 1.503	6.290 ± 1.357	5.736 ± 0.552
MONO (%)	2.18 ± 0.20	3.06 ± 1.44	3.10 ± 0.45	2.56 ± 0.96	2.38 ± 0.99
MONO (10^3/μL)	0.168 ± 0.036	0.278 ± 0.132	0.206 ± 0.047	0.190 ± 0.068	0.164 ± 0.065
EOS (%)	0.96 ± 0.25	0.80 ± 0.38	0.94 ± 0.34	1.08 ± 0.22	0.96 ± 0.40
EOS (10^3/μL)	0.072 ± 0.015	0.070 ± 0.027	0.066 ± 0.035	0.084 ± 0.034	0.066 ± 0.026
BASO (%)	0.18 ± 0.08	0.26 ± 0.09	0.18 ± 0.08	0.16 ± 0.05	0.20 ± 0.00
BASO (10^3/μL)	0.016 ± 0.009	0.024 ± 0.011	0.014 ± 0.011	0.012 ± 0.008	0.018 ± 0.004
LUC (%)	0.54 ± 0.11	0.64 ± 0.19	0.78 ± 0.33	0.76 ± 0.18	0.76 ± 0.27
LUC (10^3/μL)	0.044 ± 0.017	0.060 ± 0.016	0.054 ± 0.023	0.060 ± 0.016	0.054 ± 0.023
No. of animals	5	5	5	5	5

RBC, red blood cell; HGB, hemoglobin; HCT, hematocrit; MCV; mean corpuscular volume; MCH, mean corpuscular hemoglobin; MCHC, mean corpuscular hemoglobin concentration; RDW, red cell distribution width; HDW, hemoglobin distribution width; PLT, platelet; MPV, mean platelet volume; WBC, white blood cell; NEU, neutrophils; LYM, lymphocytes; MONO, monocytes; EOS, eosinophils; BASO, basophils; LUC, large unstained cell. Significant differences were compared with the control group, $*P < 0.05$, $**P < 0.01$, $***P < 0.001$.

Table S6. Serum biochemical values in 4 weeks repeated oral dose toxicity of WG extract in male rats

Parameters	Dose (mg/kg/day)				
	**Control**	**625**	**1250**	**2500**	**5000**
Male					
AST (IU/L)	97.42 ± 21.92	95.28 ± 15.07	102.06 ± 31.16	92.28 ± 17.53	92.54 ± 23.60
ALT (IU/L)	29.50 ± 5.41	28.58 ± 2.84	28.40 ± 5.16	30.84 ± 4.08	31.86 ± 1.57
ALP (IU/L)	196.40 ± 58.12	161.96 ± 35.08	179.58 ± 35.59	186.20 ± 18.62	181.44 ± 14.70
CPK (IU/L)	346.4 ± 136.3	394.8 ± 156.4	375.6 ± 286.4	247.4 ± 116.4	279.4 ± 145.4
TBIL (mg/dL)	0.1582 ± 0.0193	0.1552 ± 0.0325	0.1602 ± 0.0209	0.1568 ± 0.0271	0.1620 ± 0.0178
GLU (mg/dL)	122.36 ± 11.65	129.48 ± 8.25	119.68 ± 13.43	123.30 ± 10.31	133.76 ± 13.17
TCHO (mg/dL)	60.8 ± 10.2	66.6 ± 11.3	70.2 ± 17.5	58.0 ± 15.2	73.4 ± 15.0
TG (mg/dL)	71.0 ± 17.0	83.2 ± 40.3	66.8 ± 19.8	81.2 ± 25.6	93.8 ± 21.2
TP (g/dL)	6.194 ± 0.141	6.218 ± 0.267	6.208 ± 0.196	6.004 ± 0.244	6.206 ± 0.319

ALB (g/dL)	3.090 ± 0.066	3.126 ± 0.117	3.098 ± 0.061	3.078 ± 0.077	3.108 ± 0.158
A/G Ratio	0.996 ± 0.025	1.012 ± 0.048	0.998 ± 0.045	1.054 ± 0.036	1.002 ± 0.016
BUN (mg/dL)	11.78 ± 2.15	11.84 ± 0.93	11.50 ± 1.95	9.90 ± 0.99	10.16 ± 1.35
CREA (mg/dL)	0.414 ± 0.030	0.400 ± 0.030	0.388 ± 0.013	0.390 ± 0.016	0.384 ± 0.030
IP (mg/dL)	7.602 ± 0.572	7.436 ± 0.258	7.564 ± 0.220	7.660 ± 0.790	7.668 ± 0.244
Ca (mg/dL)	10.530 ± 0.260	10.638 ± 0.316	10.528 ± 0.328	10.454 ± 0.210	10.580 ± 0.171
Na (mmol/L)	141.246 ± 0.717	141.908 ± 0.788	141.740 ± 0.980	142.300 ± 0.634	141.220 ± 1.053
K (mmol/L)	4.456 ± 0.267	4.500 ± 0.111	4.488 ± 0.157	4.260 ± 0.326	4.442 ± 0.289
Cl (mmol/L)	102.658 ± 1.579	102.896 ± 1.610	102.814 ± 1.392	103.288 ± 1.769	101.274 ± 1.382
No. of animals	5	5	5	5	5

AST, aspartate aminotransferase; ALT, alanine aminotransferase; ALP, alkaline phosphatase; CPK, creatine phosphokinase; TBIL, total bilirubin; GLU, glucose; TCHO, total cholesterol; TG, triglyceride; TP, total protein; ALB, albumin; A/G, albumin/globulin; BUN, blood urea nitrogen; CREA, creatinine; IP, inorganic phosphorus; Ca, calcium; Na, sodium; K, potassium ion; Cl, chloride ion. Significant differences were compared with the control group, *$P < 0.05$, **$P < 0.01$, ***$P < 0.001$.

Table S7. Serum biochemical values in 4 weeks repeated oral dose toxicity of WG extract in female rats

Parameters	Dose (mg/kg/day)				
	Control	625	1250	2500	5000
Female					
AST (IU/L)	97.82 ± 34.52	100.54 ± 30.86	94.98 ± 20.22	84.76 ± 10.23	88.20 ± 23.25
ALT (IU/L)	23.54 ± 0.73	24.50 ± 4.37	24.84 ± 3.42	25.04 ± 2.84	24.74 ± 3.71
ALP (IU/L)	102.80 ± 22.28	109.04 ± 21.73	111.14 ± 12.37	99.34 ± 24.99	95.54 ± 18.87
CPK (IU/L)	297.8 ± 225.2	298.2 ± 193.8	230.8 ± 126.9	202.2 ± 122.1	250.2 ± 164.0
TBIL (mg/dL)	0.1794 ± 0.0079	0.1738 ± 0.0202	0.1846 ± 0.0284	0.1734 ± 0.0102	0.1750 ± 0.0264
GLU (mg/dL)	120.36 ± 18.20	112.58 ± 13.29	124.24 ± 12.88	123.90 ± 16.63	118.74 ± 15.63
TCHO (mg/dL)	69.0 ± 15.4	63.8 ± 7.4	59.4 ± 9.7	62.8 ± 18.6	58.2 ± 12.3
TG (mg/dL)	26.4 ± 4.2	29.0 ± 8.1	27.8 ± 4.3	27.6 ± 2.7	32.4 ± 8.4
TP (g/dL)	6.380 ± 0.252	6.202 ± 0.267	6.472 ± 0.391	6.328 ± 0.233	6.298 ± 0.466
ALB (g/dL)	3.390 ± 0.085	3.286 ± 0.151	3.408 ± 0.216	3.366 ± 0.116	3.308 ± 0.254
A/G Ratio	1.138 ± 0.072	1.126 ± 0.043	1.114 ± 0.047	1.138 ± 0.047	1.106 ± 0.049
BUN (mg/dL)	12.60 ± 0.58	12.82 ± 1.13	13.36 ± 1.44	11.84 ± 0.69	11.64 ± 0.97
CREA (mg/dL)	0.432 ± 0.039	0.424 ± 0.039	0.464 ± 0.025	0.434 ± 0.021	0.410 ± 0.014
IP (mg/dL)	6.146 ± 0.575	6.438 ± 0.491	6.098 ± 0.465	6.438 ± 0.419	5.928 ± 0.312
Ca (mg/dL)	10.216 ± 0.376	10.362 ± 0.349	10.132 ± 0.220	10.320 ± 0.156	10.362 ± 0.193
Na (mmol/L)	140.422 ± 1.232	140.606 ± 0.926	140.010 ± 1.510	141.082 ± 1.424	141.350 ± 1.052
K (mmol/L)	4.354 ± 0.291	4.310 ± 0.142	4.034 ± 0.096	4.236 ± 0.300	4.142 ± 0.262
Cl (mmol/L)	105.398 ± 1.826	104.218 ± 1.947	104.400 ± 0.342	104.398 ± 2.358	104.378 ± 1.494
No. of animals	5	5	5	5	5

AST, aspartate aminotransferase; ALT, alanine aminotransferase; ALP, alkaline phosphatase; CPK, creatine phosphokinase; TBIL, total bilirubin; GLU, glucose; TCHO, total cholesterol; TG, triglyceride; TP, total protein; ALB, albumin; A/G, albumin/globulin; BUN, blood urea nitrogen; CREA, creatinine; IP, inorganic phosphorus; Ca,

calcium; Na, sodium; K, potassium ion; Cl, chloride ion. Significant differences were compared with the control group, *$P < 0.05$, **$P < 0.01$, ***$P < 0.001$.

Table S8. Absolute and relative organ weights in 4 weeks repeated oral dose toxicity of WG extract in male rats

Parameters	Dose (mg/kg/day)				
	Control	625	1250	2500	5000
Male					
Body weights (g)[a)]	373.572 ± 11.268	384.114 ± 17.854	380.574 ± 21.390	377.806 ± 24.604	381.420 ± 24.088
Lung	1.555 ± 0.108	1.593 ± 0.159	1.624 ± 0.096	1.557 ± 0.080	1.552 ± 0.122
% to body weight	0.416 ± 0.025	0.415 ± 0.038	0.427 ± 0.020	0.413 ± 0.029	0.407 ± 0.018
Heart	1.335 ± 0.081	1.281 ± 0.040	1.268 ± 0.073	1.368 ± 0.152	1.295 ± 0.103
% to body weight	0.358 ± 0.030	0.334 ± 0.019	0.333 ± 0.008	0.361 ± 0.024	0.340 ± 0.022
Brain	1.951 ± 0.098	2.025 ± 0.061	2.007 ± 0.078	1.951 ± 0.074	1.975 ± 0.067
% to body weight	0.523 ± 0.031	0.528 ± 0.034	0.528 ± 0.031	0.519 ± 0.051	0.519 ± 0.023
Liver	11.525 ± 0.439	11.509 ± 1.071	11.266 ± 1.128	11.475 ± 1.328	12.819 ± 1.771
% to body weight	3.089 ± 0.179	2.992 ± 0.177	2.957 ± 0.191	3.030 ± 0.175	3.348 ± 0.281
Spleen	0.756 ± 0.126	0.777 ± 0.132	0.783 ± 0.096	0.857 ± 0.138	0.817 ± 0.111
% to body weight	0.203 ± 0.037	0.202 ± 0.029	0.205 ± 0.020	0.226 ± 0.033	0.214 ± 0.019
Kidney (left)	1.349 ± 0.113	1.394 ± 0.135	1.350 ± 0.063	1.381 ± 0.061	1.442 ± 0.131
% to body weight	0.361 ± 0.030	0.363 ± 0.032	0.355 ± 0.004	0.366 ± 0.021	0.378 ± 0.027
Kidney (right)	1.379 ± 0.118	1.433 ± 0.140	1.393 ± 0.122	1.368 ± 0.067	1.479 ± 0.133
% to body weight	0.369 ± 0.030	0.373 ± 0.031	0.366 ± 0.016	0.363 ± 0.019	0.388 ± 0.028
Thymus	0.544 ± 0.082	0.541 ± 0.186	0.455 ± 0.107	0.572 ± 0.121	0.471 ± 0.083
% to body weight	0.146 ± 0.023	0.140 ± 0.046	0.120 ± 0.030	0.151 ± 0.027	0.124 ± 0.024
No. of animals	5	5	5	5	5

[a)] Body weights were measured immediately prior to necropsy after overnight fast. Significant differences were compared with the control group, *$P < 0.05$, **$P < 0.01$, ***$P < 0.001$.

Table S9. Absolute and relative organ weights in 4 weeks repeated oral dose toxicity of WG extract in female rats

Parameters	Dose (mg/kg/day)				
	Control	625	1250	2500	5000
Female					
Body weights (g)[a)]	201.494 ± 20.373	214.142 ± 12.075	200.140 ± 14.185	201.478 ± 17.104	204.348 ± 16.211
Lung	1.113 ± 0.079	1.131 ± 0.117	1.029 ± 0.064	1.069 ± 0.111	1.121 ± 0.116
% to body weight	0.554 ± 0.030	0.527 ± 0.031	0.515 ± 0.026	0.532 ± 0.058	0.548 ± 0.034
Heart	0.757 ± 0.105	0.779 ± 0.058	0.755 ± 0.049	0.756 ± 0.060	0.822 ± 0.069
% to body weight	0.376 ± 0.043	0.363 ± 0.014	0.377 ± 0.006	0.376 ± 0.024	0.404 ± 0.045
Brain	1.797 ± 0.088	1.778 ± 0.113	1.747 ± 0.138	1.783 ± 0.033	1.799 ± 0.139

% to body weight	0.898 ± 0.089	0.831 ± 0.041	0.877 ± 0.090	0.890 ± 0.070	0.882 ± 0.054
Liver	5.834 ± 0.502	5.891 ± 0.292	5.524 ± 0.428	5.545 ± 0.497	5.758 ± 0.458
% to body weight	2.900 ± 0.116	2.752 ± 0.057	2.760 ± 0.102	2.756 ± 0.195	2.821 ± 0.169
Spleen	0.438 ± 0.115	0.520 ± 0.034	0.429 ± 0.046	0.440 ± 0.067	0.450 ± 0.064
% to body weight	0.219 ± 0.058	0.243 ± 0.015	0.214 ± 0.015	0.217 ± 0.016	0.220 ± 0.018
Kidney (left)	0.755 ± 0.052	0.797 ± 0.049	0.703 ± 0.077	0.733 ± 0.077	0.779 ± 0.035
% to body weight	0.377 ± 0.039	0.372 ± 0.017	0.351 ± 0.030	0.364 ± 0.022	0.383 ± 0.025
Kidney (right)	0.767 ± 0.062	0.805 ± 0.073	0.751 ± 0.090	0.740 ± 0.061	0.812 ± 0.032
% to body weight	0.383 ± 0.046	0.376 ± 0.024	0.375 ± 0.034	0.367 ± 0.017	0.399 ± 0.025
Thymus	0.388 ± 0.040	0.486 ± 0.083	0.311 ± 0.081	0.380 ± 0.081	0.356 ± 0.091
% to body weight	0.193 ± 0.020	0.227 ± 0.036	0.155 ± 0.037	0.189 ± 0.038	0.174 ± 0.040
No. of animals	5	5	5	5	5

[a)] Body weights were measured immediately prior to necropsy after overnight fast. Significant differences were compared with the control group, $*P < 0.05$, $**P < 0.01$, $***P < 0.001$.

Table S10. Necropsy findings in 4 weeks repeated oral dose toxicity of WG extract in male rats

Parameters	Observation Type	Dose (mg/kg/day)				
		Control	625	1250	2500	5000
Male						
Adrenal Gland	Normal	5	5	5	5	5
Aorta	Normal	5	5	5	5	5
Bone Marrow, Sternum	Normal	5	5	5	5	5
Brain	Submitted	5	5	5	5	5
	Normal	5	5	5	5	5
Cervix	Normal	-	-	-	-	-
Coagulating Gland	Normal	5	5	5	5	5
Epididymis	Normal	5	5	5	5	5
Esophagus	Normal	5	5	5	5	5
Eye with Optic Nerve	Normal	5	5	5	5	5
Harderian Gland	Normal	5	5	5	5	5
Heart	Submitted	5	5	5	5	5
	Normal	5	5	5	5	5
Cecum	Normal	5	5	5	5	5
Colon	Normal	5	5	5	5	5
Duodenum	Normal	5	5	5	5	5
Ileum	Normal	5	5	5	5	5
Jejunum	Normal	5	5	5	5	5
Rectum	Normal	5	5	5	5	5
Kidney	Submitted	5	5	5	5	5

	Normal	5	4	5	5	5
	Hydronephrosis: right	0	1	0	0	0
Liver	Submitted	5	5	5	5	5
	Normal	5	5	5	5	5
Lung	Submitted	5	5	5	5	5
	Normal	5	5	5	5	5
Lymph Node, Mesenteric	Normal	5	5	5	5	5
Lymph Node, Mandibular	Normal	5	5	5	5	5
Skeletal Muscle	Normal	5	5	5	5	5
Nerve, Peripheral	Normal	5	5	5	5	5
Ovary	Normal	-	-	-	-	-
Pancreas	Normal	5	5	5	5	5
Parathyroid Gland	Normal	5	5	5	5	5
Pituitary Gland	Normal	5	5	5	5	5
Prostate Gland	Normal	5	5	5	5	5
Salivary Gland	Normal	5	5	5	5	5
Seminal Vesicle	Normal	5	5	5	5	5
Skin, Mammary	Normal	5	5	5	5	5
Spinal Cord, Cervical	Normal	5	5	5	5	5
Spinal Cord, Thoracic	Normal	5	5	5	5	5
Spinal Cord, Lumbar	Normal	5	5	5	5	5
Spleen	Submitted	5	5	5	5	5
	Normal	5	5	5	5	5
Stomach	Normal	5	5	5	5	5
Testis	Normal	5	5	5	5	5
Thymus	Submitted	5	5	5	5	5
	Normal	5	5	5	5	5
Thyroid Gland	Normal	5	5	5	5	5
Tongue	Normal	5	5	5	5	5
Trachea	Normal	5	5	5	5	5
Urinary Bladder	Normal	5	5	5	5	5
Uterus	Normal	-	-	-	-	-
Vagina	Normal	-	-	-	-	-
Femorotibial Joint	Normal	5	5	5	5	5
No. of animals		5	5	5	5	5

-: Not applicable.

Table S11. Necropsy findings in 4 weeks repeated oral dose toxicity of WG extract in female rats

Parameters	Observation Type	Dose (mg/kg/day)				
		Control	625	1250	2500	5000

Female						
Adrenal Gland	Normal	5	5	5	5	5
Aorta	Normal	5	5	5	5	5
Bone Marrow, Sternum	Normal	5	5	5	5	5
Brain	Submitted	5	5	5	5	5
	Normal	5	5	5	5	5
Cervix	Normal	5	5	5	5	5
Coagulating Gland	Normal	-	-	-	-	-
Epididymis	Normal	-	-	-	-	-
Esophagus	Normal	5	5	5	5	5
Eye with Optic Nerve	Normal	5	5	5	5	5
Harderian Gland	Normal	5	5	5	5	5
Heart	Submitted	5	5	5	5	5
	Normal	5	5	5	5	5
Cecum	Normal	5	5	5	5	5
Colon	Normal	5	5	5	5	5
Duodenum	Normal	5	5	5	5	5
Ileum	Normal	5	5	5	5	5
Jejunum	Normal	5	5	5	5	5
Rectum	Normal	5	5	5	5	5
Kidney	Submitted	5	5	5	5	5
	Normal	5	5	5	5	5
	Hydronephrosis: right	0	0	0	0	0
Liver	Submitted	5	5	5	5	5
	Normal	5	5	5	5	5
Lung	Submitted	5	5	5	5	5
	Normal	5	5	5	5	5
Lymph Node, Mesenteric	Normal	5	5	5	5	5
Lymph Node, Mandibular	Normal	5	5	5	5	5
Skeletal Muscle	Normal	5	5	5	5	5
Nerve, Peripheral	Normal	5	5	5	5	5
Ovary	Normal	5	5	5	5	5
Pancreas	Normal	5	5	5	5	5
Parathyroid Gland	Normal	5	5	5	5	5
Pituitary Gland	Normal	5	5	5	5	5
Prostate Gland	Normal	-	-	-	-	-
Salivary Gland	Normal	5	5	5	5	5
Seminal Vesicle	Normal	-	-	-	-	-
Skin, Mammary	Normal	5	5	5	5	5
Spinal Cord, Cervical	Normal	5	5	5	5	5

Spinal Cord, Thoracic	Normal	5	5	5	5	5
Spinal Cord, Lumbar	Normal	5	5	5	5	5
Spleen	Submitted	5	5	5	5	5
	Normal	5	5	5	5	5
Stomach	Normal	5	5	5	5	5
Testis	Normal	-	-	-	-	-
Thymus	Submitted	5	5	5	5	5
	Normal	5	5	5	5	5
Thyroid Gland	Normal	5	5	5	5	5
Tongue	Normal	5	5	5	5	5
Trachea	Normal	5	5	5	5	5
Urinary Bladder	Normal	5	5	5	5	5
Uterus	Normal	5	5	5	5	5
Vagina	Normal	5	5	5	5	5
Femorotibial Joint	Normal	5	5	5	5	5
No. of animals		5	5	5	5	5

-: Not applicable.
